# Polysaccharides from the root tubers of *Curcuma wenyujin* ameliorate cholestatic liver injury associated with remodeling gut microbiota and restoring bile acid homeostasis

**DOI:** 10.3389/fphar.2026.1847544

**Published:** 2026-09-03

**Authors:** Juan Zou, Yuxin Kan, Wenqi Song, Shuyu Song, Doudou Huang, Jingwen Liu, Fujiang Guo

**Affiliations:** 1 School of Pharmacy, Shanghai University of Traditional Chinese Medicine, Shanghai, China; 2 Experiment Center for Science and Technology, Shanghai University of Traditional Chinese Medicine, Shanghai, China; 3 Shanghai Seventh People’s Hospital, Shanghai University of Traditional Chinese Medicine, Shanghai, China

**Keywords:** bile acid, cholestasis, *Curcuma aromatica Salisb.*, *Curcuma wenyujin*, gut microbiota, polysaccharides

## Abstract

**Background:**

The root tuber of *Curcuma wenyujin* Y. H. Chen et C. Ling (taxonomically a synonym of the accepted species *Curcuma aromatica Salisb*.; hereafter *C. wenyujin*) has been prescribed in Chinese medicine for over a millennium to promote bile flow and alleviate jaundice. While its lipophilic constituents have received considerable attention, the bioactive potential of its water-soluble polysaccharides remains unclear.

**Methods:**

A polysaccharide-enriched fraction (CWP) was isolated from *C. wenyujin* by hot-water extraction followed by enzymatic starch removal and dialysis. Its structural features were characterized by high performance gel permeation chromatography (HPGPC), high-performance anion-exchange chromatography with a pulsed amperometry detector (HPAEC-PAD), fourier transform infrared (FT-IR), and proton nuclear magnetic resonance (^1^H NMR). Hepatoprotective effects were evaluated in an α-naphthyl isothiocyanate (ANIT)-induced cholestasis model in mice. Underlying mechanisms were investigated using label-free hepatic proteomics, 16S rRNA gene sequencing, and targeted bile acid profiling by liquid chromatograph mass spectrometer (LC-MS/MS).

**Results:**

CWP was characterized as a mannose- and glucose-rich polysaccharide-enriched fraction (average Mw 5.89 kDa; mannose 47.05 mol%, glucose 33.67 mol%) containing both α- and β-glycosidic configurations. Oral administration of CWP significantly reduced serum alanine aminotransferase (ALT), aspartate aminotransferase (AST), alkaline phosphatase (ALP), and total bile acids (TBA) at both doses tested, and attenuated hepatocyte necrosis and inflammatory infiltration in ANIT-challenged mice. Mechanistically, CWP upregulated bile acid synthesis enzymes (*Cyp27a1*), efflux transporters (*Mrp2/3/4*), and detoxification enzymes (*Ugt1a1*, *Cyp3a11*), leading to a reduction in hepatotoxic Tauro-β-muricholic acid (T-β-MCA) (from 22.0% to 14.6%) and an increase in cytoprotective ursodeoxycholic acid (UDCA). In the gut, CWP selectively suppressed *Enterobacter* while enriching *Lactobacillus* and *Adlercreutzia*, changes that correlated with the improved bile acid profile. CWP also inhibited hepatic NF-κB activation and neutrophil infiltration, and restored ileal tight-junction proteins (ZO-1, Occludin).

**Conclusion:**

CWP alleviates ANIT-induced cholestatic liver injury by restoring hepatic bile acid clearance, selectively remodeling the gut microbiota, and suppressing inflammation across the gut-liver axis. These results support further development of CWP as a functional macromolecule for managing cholestatic liver diseases.

## Graphical Abstract

**Figure:**
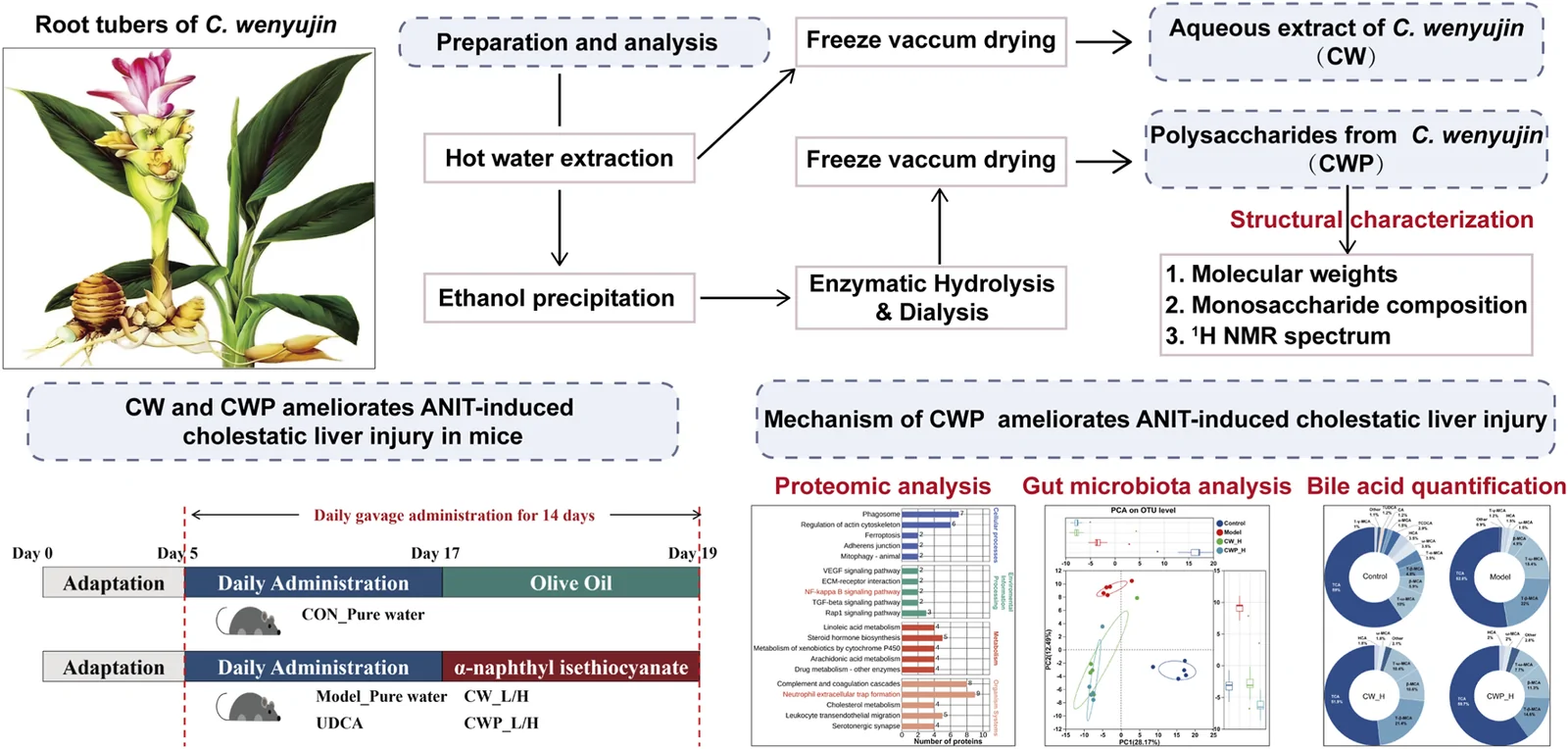

## 1 Introduction

Cholestasis is a pathological condition characterized by impaired bile flow resulting from hepatocellular secretory dysfunction or bile duct obstruction (Hilscher et al., 2020). Early diagnosis primarily relies on biochemical markers, including elevated alkaline phosphatase (ALP), γ-glutamyl transferase (γ-GT) and conjugated bilirubin levels (European, 2009). Prolonged cholestatic conditions often lead to oxidative stress, persistent inflammation, and progressive liver injury, which significantly impact nutritional status and overall quality of life (Banales et al., 2019). Ursodeoxycholic acid (UDCA) remains the first line therapy, yet approximately 40% of patients fail to achieve adequate biochemical response (Cabrera et al., 2019). Nuclear receptor targeting: Obeticholic acid (OCA), a farnesoid X receptor (FXR) agonist, improves cholestatic parameters but causes dose limiting pruritus in up to 70% of recipients (Nevens et al., 2016). These therapeutic gaps have prompted a search for alternative interventions, particularly bioactive natural products with multi-target pharmacological profiles.

The pathogenesis of cholestasis is driven by a feedback loop between bile acid accumulation and hepatic inflammation (Gujral et al., 2003). Retained bile acids, principally chenodeoxycholic acid (CDCA) and cholic acid (CA) induce mitochondrial dysfunction, oxidative stress, and hepatocyte apoptosis (Tong et al., 2025; Mosaoa et al., 2024; Schoemaker et al., 2003). The resulting cellular damage activates NF-κB signaling and upregulates pro-inflammatory cytokines (TNF-α, IL-1β, and IL-6), which in turn suppress bile acid synthesis enzymes (CYP7A1 and CYP8B1) and canalicular efflux transporters (BSEP and MRP2), further impairing bile acid clearance (Allen et al., 2011; Zollner et al., 2003). This cycle is amplified by bidirectional communication along the gut-liver axis. Under physiological conditions, intestinal bacteria particularly *Clostridium* and *Bacteroides* species convert primary bile acids to secondary metabolites via 7α-dehydroxylation and deconjugation (Rimal et al., 2024; Fuchs et al., 2025). Cholestasis disrupts this homeostasis: reduced biliary output to the intestinal lumen promotes dysbiosis, alters the bile acid pool composition, compromises tight-junction integrity, and facilitates bacterial translocation and endotoxemia (Thibaut and Bindels, 2022; Liu et al., 2024; Chang et al., 2025). These observations point to the gut-liver axis as a promising multi-level therapeutic target.

*Curcumae Radix*, the dried root tuber of *Curcuma wenyujin* Y. H. Chen et C. Ling, C. *longa* L., *C. kwangsiensis* S. G. Lee et C. F. Liang, or *C. phaeocaulis* Val. has been used in traditional Chinese medicine for over 1,000 years to promote bile flow, resolve blood stasis, and alleviate jaundice (Li et al., 2021). Among these botanical sources, *Curcuma. wenyujin* Y. H. Chen et C. Ling (*C*. *wenyujin*) is the most widely prescribed. Phytochemical studies have characterized diverse constituents including sesquiterpenoids, volatile oils, and polysaccharides (Li et al., 2021; Lee et al., 2025; Wang et al., 2021). Previous work has focused on lipophilic components, however, water-soluble polysaccharides which is the predominant constituents extracted during traditional aqueous decoction remain largely unexplored. Because plant derived polysaccharides are increasingly recognized as modulators of the gut-liver axis through prebiotic and immunomodulatory activities, they represent compelling candidates for investigation in the context of cholestasis.

In this study, we used an integrated approach combining pharmacological evaluation, structural characterization, hepatic proteomics, 16S rRNA gene sequencing, and targeted bile acid metabolomics to systematically investigate the therapeutic potential of *C. wenyujin* in α-naphthyl isothiocyanate (ANIT)-induced cholestasis. Specifically, our objectives were to validate the hepatoprotective efficacy of the crude aqueous extract (CW), evaluate the hepatoprotective potential of a polysaccharide-enriched fraction from *C. wenyujin* (CWP), and investigate the potential molecular and microbial pathways associated with CWP mediated hepatoprotection. We hypothesized that CWP pretreatment ameliorates cholestatic injury through modulation of hepatic bile acid metabolism, intestinal microbiota composition, and inflammatory signaling along the gut-liver axis.

## 2 Materials and methods

### 2.1 Materials and reagents

The root tubers of *C. wenyujin* were cultivated and collected from Lishui city, Zhejiang Province of China (GPS: 27^◦^54′N, 119^◦^0′E), in March 2024, and identified by Dr. Yingchun Wu (Associate Professor, School of Pharmacy, Shanghai University of Traditional Chinese Medicine) according to the morphological and microscopic criteria of the Chinese Pharmacopoeia (2020 Edition, Volume I, monograph “*Curcumae Radix*”). A retained botanical reference sample (No. 20240322) consisting of tuberous root material and extraction has been deposited in the −20 °C freezer in Laboratory 1309, Shanghai University of Traditional Chinese Medicine; Ursodeoxycholic acid (UDCA, 250 mg/capsule) was purchased from Ursofalk. Alpha-naphthylisothiocyanate (ANIT, purity ≥95%) was obtained from Sigma-Aldrich. Olive oil was purchased from Aladdin. anti-NF-κB p65 (8242S, 1:1000), anti-p-NF-κB p65 (3033S, 1:1000), were purchased from Cell Signaling Technology; anti-IL-1β (26048-1-AP, 1:1000), was purchased from Proteintech; anti-GAPDH (A10139, 1:2000), HRP-goat anti-rabbit (R00001, 1:10000), HRP-goat anti-mouse antibodies (M00001, 1:10000) were purchased from Nature Biosciences; anti-TNF-α (A24214, 1:1000), anti-ZO-1(YM8448, 1:2000) and anti-Occludin (66378-Ig, 1:5000) were purchased from ABclonal Technology.

### 2.2 Preparation of CW and CWP from *Curcuma wenyujin*

CW (aqueous extract derived from *C. wenyujin*): The steam-dried and powdered root tubers (500 g) were refluxed twice in 10- and 8- fold volumes of water (2 h and 1 h, respectively). The combined filtrates were evaporated to dryness *in vacuo* at 60 °C to yield the powdered aqueous extract. The drug extract ratio (DER) was 7.8:1 (500 g of dried root tubers yielded 64 g of dried extract). To ensure chemical consistency and traceability, eight characteristic sesquiterpenoid metabolites in the botanical drug were quantified by HPLC. The analysis verified the stable chemical profile of the raw material. Detailed analytical methods, chromatograms, and quantitative results are provided in the (Supplementary Figure S1; Supplementary Table S1; Supplementary Table S2).

CWP (polysaccharide-enriched fraction derived from *C. wenyujin*): The steam-dried and powdered root tubers (2.0 kg) were extracted twice with boiling distilled water (1:10 and 1:20, w/v). The combined filtrates were concentrated and precipitated with ethanol to a final concentration of 80% (v/v) at 4 °C overnight. The precipitate was collected by centrifugation (4000 rpm, 10 min), washed with 95% ethanol, redissolved in water and lyophilized to obtain crude polysaccharides. The crude product was redissolved and treated with α-amylase (0.2%, w/w) at 60 °C for 30 min until a negative iodine test confirmed complete starch removal. The de-starched solution was lyophilized, redissolved in pure water, and subjected to repeated freeze thaw cycles to remove proteins. The resulting supernatant was dialyzed against distilled water (MWCO: 3500 Da) for 72 h and finally lyophilized to yield purified CWP.

### 2.3 Animal experiments

Specific pathogen-free (SPF) male C57BL/6J mice 6–8 weeks old, weighing 22–24 g was purchased from SLAC Laboratory Animal Co., Ltd. (Shanghai, China), and were housed in the Laboratory Animal Center of Shanghai University of Traditional Chinese Medicine. Throughout the experiment, the environmental conditions were maintained on a 12-h light-dark cycle, the temperature was kept at 20 °C–22 °C, and relative humidity was controlled at 60%–70%. All animal experiments were authorized by the Animal Committee of Shanghai University of Traditional Chinese Medicine (PZSHUTCM2403110007; PZSHUTCM2507030005).

Experiment I: After 1 week of acclimatization, animals were randomly divided into five groups (n = 8); control, model; the aqueous extract from *C. wenyujin* with low and high dose (CW_L, 60 mg/kg, equals to 0.75 g raw material/kg; CW_H, 120 mg/kg, equals to 1.5 g raw material/kg), and ursodeoxycholic acid (UDCA, 100 mg/kg). At 9:00 a.m. daily (once a day), the Control and Model groups received pure water via oral gavage, while the other three groups (CW_L, CW_H, and UDCA) received their respective treatments via oral gavage for 14 consecutive days. The gavage volume for all treatments was strictly standardized to 0.1 mL/kg based on daily recorded body weights. On day 12, the Control group was administered an equivalent amount of olive oil via oral gavage. The remaining groups were administered 50 mg/kg ANIT (dissolved in olive oil, at a volume of 0.1 mL/kg) via oral gavage to establish the model (Yang et al., 2026; He et al., 2024). 48 h after ANIT administration, mice were anesthetized and euthanized. Serum and liver tissue samples were collected.

Experiment II: Animals (same strain, sex, and age as described in Experiment I) were randomly divided into six groups (n = 8); control, model; the aqueous extract from *C. wenyujin* with high dose (CW_H, 120 mg/kg), CWP low and high dose (CWP_L, 50 mg/kg, CWP_H, 100 mg/kg) and ursodeoxycholic acid (UDCA, 100 mg/kg). The establishment of the animal model, the daily gavage procedures (once daily, volume of 0.1 mL/kg), and subsequent sampling processing were performed identically as described in Experiment I.

### 2.4 Sample collection

At the end of the experiments, mice were completely anesthetized via inhalation of 4% isoflurane. Blood samples were collected from the retro orbital venous plexus into sterile tubes. The blood was allowed to stand at room temperature for 30 min to clot, followed by centrifugation at 8000 rpm for 15 min. The upper serum layer was collected; a fraction was used for immediate biochemical analysis, and the remainder was stored at −80 °C (Regarding sample sizes, n = 8 samples per group were used in Experiment II. In Experiment I, individual serum samples with observed hemolysis or insufficient volume were excluded, resulting in n = 7).

Following blood collection, liver, ileum, and fecal samples were harvested. To ensure anatomical consistency, liver tissues were strictly harvested from the identical left hepatic lobe. The lobe was immediately bisected: one portion was fixed in 4% paraformaldehyde for tissue sectioning (H&E staining and immunofluorescence), and the other portion was snap-frozen in liquid nitrogen. Due to the limited tissue mass obtained from a single lobe, the frozen portions were systematically allocated: 5 samples were used for proteomics and qRT-PCR (n = 5), and the remaining 3 were used exclusively for Western blot (n = 3).

For intestinal analyses, formed fecal pellets within the ileal lumen were gently extruded using sterile forceps into sterile tubes, immediately snap-frozen, and stored at −80 °C for 16S rDNA sequencing (n = 5). Subsequently, the corresponding ileal tissue segments (approximately 5 cm proximal to the ileocecal valve) were harvested, washed with ice-cold PBS, and processed for H&E staining and Western blot analysis (n = 3).”

### 2.5 Serum biochemical and histological evaluation

Serum samples from mice were analyzed for alanine aminotransferase (ALT), aspartate aminotransferase (AST), alkaline phosphatase (ALP), total bilirubin (TBIL), direct bilirubin (DBIL), and total bile acid (TBA) using a Hitachi 3500 Automated Biochemical Analyzer (Hitachi High-Technologies, Japan).

Liver and ileum tissue samples were fixed in 4% paraformaldehyde solution. Subsequent processing including paraffin embedding, sectioning, hematoxylin and eosin (H&E) staining, and whole-slide digital scanning was performed by Yangming Medical Laboratory Co., Ltd (Ningbo, China). Liver necrosis was evaluated using a classic 0 to 4 semi-quantitative scoring system based on the percentage of the lesion area: 0, normal (no necrosis or inflammation); 1, mild (<25%); 2, moderate (25%–50%); 3, severe (50%–75%); 4, very severe (>75%). Intestinal villus height was measured as the vertical distance from the villus tip to the crypt junction using ImageJ software.

### 2.6 Real-time PCR and western blotting assay

Total RNA was extracted from liver tissues using TRIzol reagent (Takara, Japan) and reverse-transcribed into cDNA. Quantitative real-time PCR was performed, and relative mRNA expression was calculated using the 2^−ΔΔCT^ method with *Gapdh* as the internal control. Detailed primers are provided in the Supplementary Materials (Supplementary Table S3).

Liver tissues were homogenized in RIPA lysis buffer containing 1% PMSF (Beyotime, China). Total proteins (30 μg/lane) were separated by SDS-PAGE, transferred to membranes, sequentially incubated with primary (4 °C, overnight) and corresponding secondary antibodies, and visualized via chemiluminescence. General procedures for these assays were performed as previously described (Li et al., 2019).

### 2.7 Proteomic sample pretreatment and analysis

Liver tissue samples (50 mg) were collected from mice in the Control, Model, and CW_H groups. The tissues were homogenized in Tissue Lysis Buffer supplemented with protease inhibitor cocktail using a mechanical tissue homogenizer. Following centrifugation, the supernatant was collected, and total protein concentration was determined using a commercial quantification kit. Subsequently, aliquots containing 100 µg of protein per sample were reduced and alkylated with dithiothreitol and iodoacetamide, subjected to followed by tryptic digestion (enzyme-to-protein ratio 1:50, w/w) at 37 °C overnight. The resulting peptides were desalted using Sep-Pak C18 cartridges.

Peptides were separated on a self-packed C18 reverse-phase column (75 μm × 250 mm; 1.9 μm) using a nano-flow LC system. Mobile phase A consisted of water and mobile phase B consisted of acetonitrile. Peptides were eluted at a flow rate of 500 nL/min with an injection volume of 6 μL, using the following gradient program: 0–5 min, 5% B; 5–90 min, 5%–25% B (linear); 90–105 min, 25%–35% B (linear); 105–110 min, 35%–80% B; 110–115 min, 80% B; 115–120 min, 80%–5% B. Mass spectrometric detection was performed on an Orbitrap Eclipse Tribrid Mass Spectrometer (Thermo Fisher Scientific). The electrospray ionization voltage was set at 2.1 kV. MS1 full scans were acquired in the Orbitrap over a mass range of m/z 400–1600. For protein identification, the generated raw mass spectrometry data were processed using Spectronaut software (Biognosys, Schlieren, Switzerland). The MS raw data were searched against mouse UniProt database (containing 21852 target sequences). To ensure high confidence in protein identification, the false discovery rate (FDR) thresholds for both peptide spectrum matches (PSMs) and proteins were strictly set to <1% (FDR <0.01).

### 2.8 Chemical characterization of CWP

Molecular weights of the polysaccharide sample were estimated by high performance gel permeation chromatography (HPGPC). The standard curve was established using different pullulans with known molecular weight (P-5, P-10, P-20, P-50, P-100, P-200, P-400 and P-800). The molecular weight of the polysaccharide sample was calculated by comparing its elution time with the standard calibration curve using Agilent GPC software.

Fourier transform infrared (FT-IR) spectroscopy analysis of polysaccharide sample was analyzed using the KBr pellet method. Briefly, the sample was thoroughly ground and homogeneously mixed with potassium bromide (KBr) powder at a 1:100 ratio, and subsequently pressed into a pellet. The IR spectra were recorded on an IRAffinity-1S spectrometer (Shimadzu, Japan) over a scanning range of 4,000–400 cm^-1^.

The monosaccharide composition was analyzed by high-performance anion-exchange chromatography with a pulsed amperometry detector (HPAEC-PAD) following acid hydrolysis. Briefly, the sample (approx. 5 mg) was hydrolyzed with 2 M trifluoroacetic acid at 121 °C for 2 h in a sealed vial. After cooling, the hydrolysate was dried under a stream of nitrogen, and residual TFA was thoroughly removed by co-distillation with methanol (3 cycles). Data acquisition and processing were managed by Chromeleon 7.2 CDS software. Monosaccharides were identified and quantified by comparing their retention times and peak areas against those of authentic standards run under the identical conditions. The analysis was performed on a Thermo Scientific Dionex ICS-5000+ system equipped with a CarboPac PA-20 anion-exchange column (3 × 150 mm). The calibration curves and corresponding regression coefficients for monosaccharide standards are presented in Supplementary Table S4.

Proton nuclear magnetic resonance (^1^H-NMR) analysis: 2 mg of the polysaccharide sample was accurately weighed, mixed with 72% sulfuric acid, and incubated overnight at room temperature. Following the addition of 200 μL of deuterium oxide (D_2_O), the mixture was sealed in a glass ampoule and hydrolyzed in an oven at 100 °C for 5 h. After cooling to room temperature, 10 μL of concentrated sulfuric acid and 10 μL of maleic acid internal standard solution were added. The mixture was then centrifuged, and 200 μL of the resulting supernatant was transferred into a standard NMR tube. The ^1^H-NMR spectrum was recorded using a Bruker Biospin 600 MHz NMR spectrometer.

### 2.9 Gut microbiota profiling and analysis

Total microbial genomic DNA was extracted from ileal fecal samples using the FastPure Stool DNA Isolation Kit (MJYH, Shanghai, China). The V3-V4 hypervariable region of the 16S rRNA gene was amplified using the primer pair 338F (5′-ACT​CCT​ACG​GGA​GGC​AGC​AG-3′) and 806R (5′-GGACTACHVGGGTWTCTAAT-3′). The PCR program consisted of an initial denaturation at 95 °C for 3 min, followed by 27 cycles of 95 °C for 30 s, 55 °C for 30 s, and 72 °C for 45 s, with a final extension at 72 °C for 10 min. Purified amplicons were sequenced on an Illumina NextSeq 2000 platform (Illumina, San Diego, USA).

Raw sequencing data were filtered by fastp version 0.19.6 and merged by FLASH version 1.2.11. Reads with an average quality score <20 over a 50 bp sliding window were discarded, and merged sequences with >2 bp mismatches were removed. Chimera detection and OTU clustering were performed simultaneously using UPARSE (version 7.1) at a 97% similarity threshold. Taxonomy was assigned using the RDP Classifier algorithm against the SILVA 16S rRNA database (release SSU123) with a confidence threshold of 70%.

### 2.10 Quantification of bile acid in the liver

Liver tissue samples (25 mg) were homogenized in 350 μL of 80% methanol containing 50 μL of internal standard working solution (CDCA-D4, 200 ng/mL). After low-temperature disruption and centrifugation, the supernatant was evaporated to dryness under nitrogen and reconstituted in 100 μL of 50% acetonitrile. Mixed bile acid calibration standards were prepared by serial dilution in 50% acetonitrile and processed identically to study samples. Analyses were performed on an AB SCIEX QTRAP 6500+ UHPLC-MS/MS system operating in negative ion mode. Calibration curves and the relative standard deviation (RSD) values of quality control (QC) samples are presented in (Supplementary Table S6). The mobile phases and conditions of liquid chromatography and mass spectrometry were the same as those in our previous studies (Zou et al., 2023; Wu et al., 2017).

### 2.11 Statistical analysis

All data are expressed as the mean ± standard error of the mean (SEM). Statistical analyses were performed using GraphPad Prism (version 1.1.37.01) and IBM SPSS Statistics software. For multi-group comparisons, one-way analysis of variance (one-way ANOVA) was applied. Prior to *post hoc* testing, variance homogeneity across groups was assessed using Levene’s test. When Levene’s test indicated homogeneity of variance (*p* > 0.05), pairwise comparisons were performed using the LSD *post hoc* test. When variances were found to be unequal (*p* < 0.05), Dunnett’s T3 test was used instead. For 16S rRNA sequencing data, genus-level differential abundance among groups was assessed using the Kruskal-Wallis test (*p* < 0.05). For proteomic data, differential protein expression was determined by one-way ANOVA with Benjamini–Hochberg (BH) correction for multiple comparisons; proteins with an adjusted *p* < 0.05 and fold change ≥1.5 were considered differentially expressed. For all other comparisons, *p* < 0.05 was considered statistically significant.

## 3 Results

### 3.1 CW ameliorates ANIT-induced cholestatic liver injury

**FIGURE 1:**
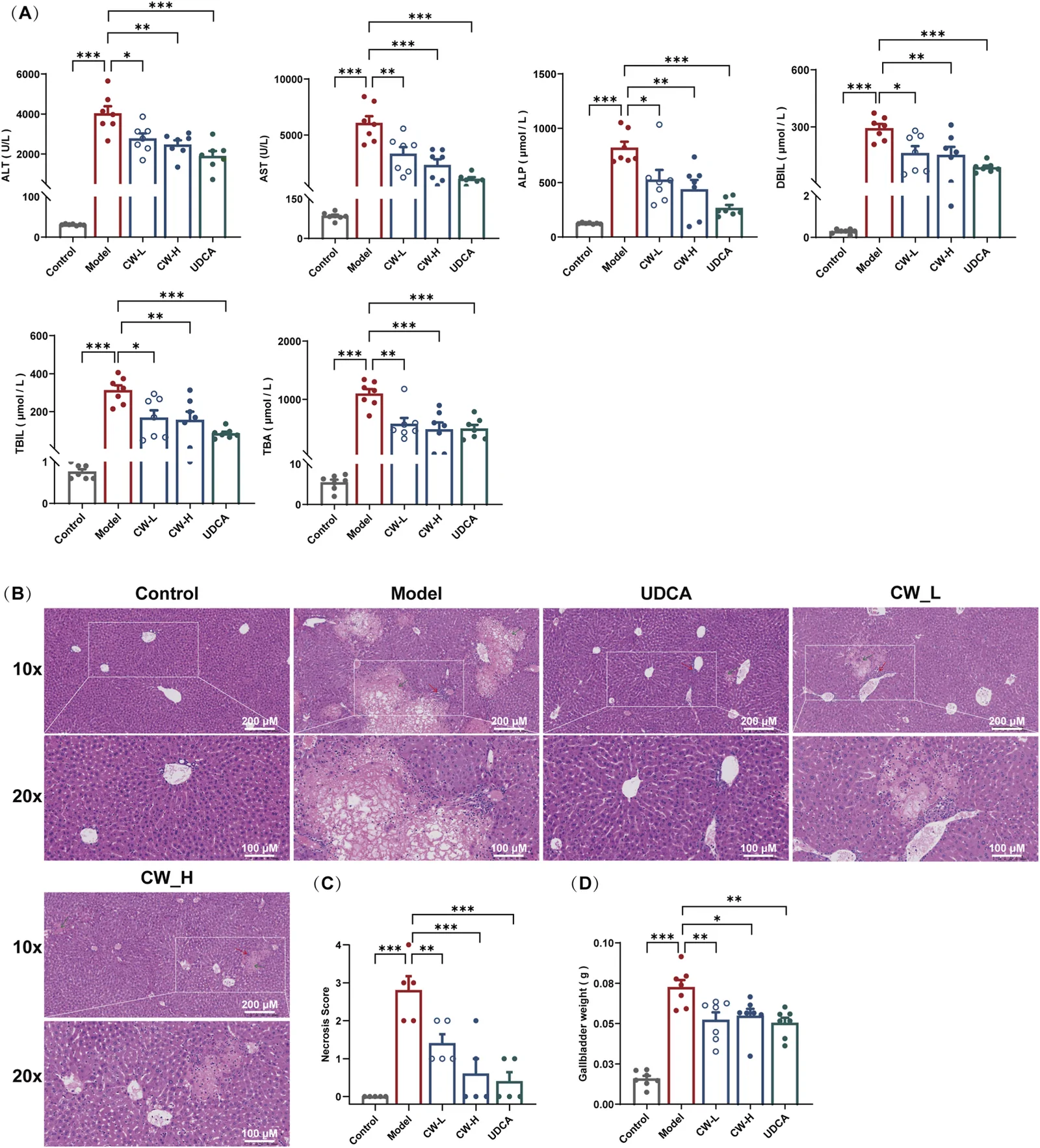
CW ameliorates ANIT-induced cholestatic liver injury in mice. **(A)** Serum biochemical parameters (ALT, AST, ALP, DBIL, TBIL and TBA) following *Curcuma wenyujin* aqueous extract (CW) treatment. **(B)** Representative H&E-stained liver tissue sections showing histopathological changes (Magnification: ×10, scale bar = 200 μm; 20×, scale bar = 100 μm). **(C)** Histopathological necrosis score based on H&E staining. **(D)** Quantitative analysis of gallbladder weight. Data are presented as mean ± SEM (n = 7 per group for serum and gallbladder weights; n = 5 for necrosis score). Statistical significance was determined by one-way ANOVA (*p < 0.05, **p < 0.01, ***p < 0.001 vs. Model group).

ANIT administration induced severe cholestatic liver injury, as evidenced by significant elevations of serum hepatocellular damage markers (ALT and AST) and cholestasis biomarkers (ALP, TBIL, DBIL, and TBA) compared to the control group. Both CW_L and CW_H treatments significantly attenuated these biochemical alterations compared to the Model group, with CW_H showing numerically greater reductions across most parameters (Figure 1A).

Histopathological examination revealed characteristic features of cholestatic liver injury in the model group, including extensive hepatocyte necrosis and prominent inflammatory cell infiltration. CW administration markedly ameliorated these pathological changes, with CW_H showing a greater degree of histological improvement (Figures 1B,C). Additionally, CW intervention effectively reduced ANIT-induced gallbladder enlargement, as demonstrated by decreased gallbladder weight (Figure 1D).

### 3.2 Proteomic profiling reveals multi-target regulation by CW

**FIGURE 2:**
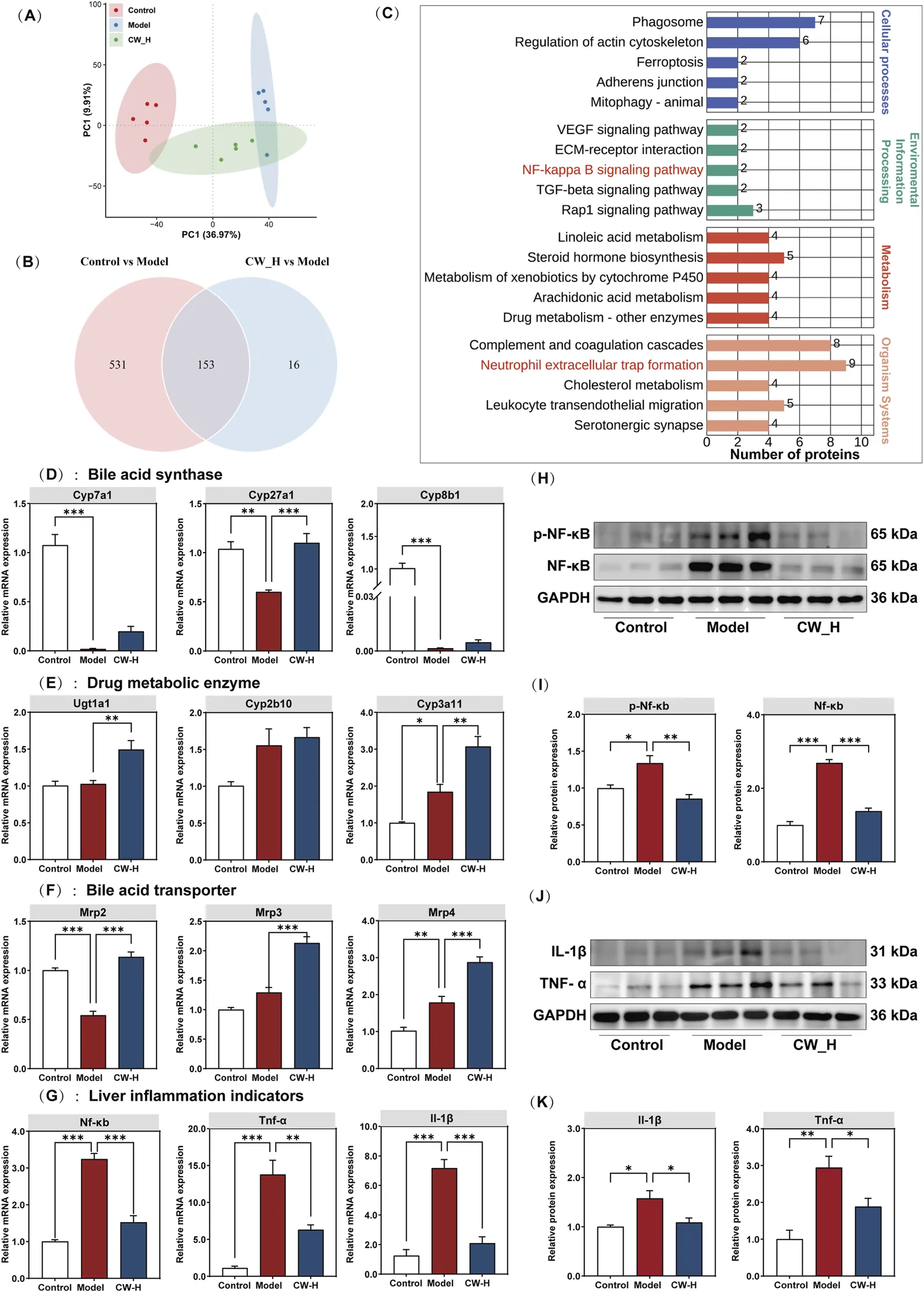
Proteomic analysis reveals hepatoprotective mechanisms of CW against cholestatic liver injury. **(A)** Principal component analysis (PCA) plot of proteomic profiles distinguishing Control, Model, and CW_H groups. **(B)** Venn diagrams illustrating the overlap of differentially expressed proteins between Control vs. Model (left) and CW_H vs. Model (right). **(C)** KEGG pathway enrichment analysis of differentially expressed proteins. **(D–G)** Relative mRNA expression levels of hepatic genes involved in **(D)** bile acid synthesis, **(E)** drug metabolic enzyme, **(F)** bile acid transport, and **(G)** liver inflammation indicators, normalized to GAPDH (n = 5). **(H,I)** Representative Western blot images and quantitative analysis of NF-κB and phosphorylated NF-κB (p-NF-κB) in liver tissue. **(J,K)** Representative Western blot images and quantitative analysis of IL-1β and TNF-α in liver tissue. Data are presented as mean ± SEM. Statistical significance was assessed by one-way ANOVA (*p < 0.05, **p < 0.01, ***p < 0.001 vs. model group).

To systematically elucidate the molecular mechanisms underlying CW’s hepatoprotective effects, we performed label-free quantitative proteomic analysis of liver tissues. High-resolution LC-MS/MS analysis identified and quantified 4,242 non-redundant proteins across all experimental group (Supplementary Figure S2). Principal component analysis (PCA) demonstrated clear separation between the control and model groups along the first principal component, indicating profound proteomic dysregulation induced by cholestatic injury (Figure 2A).

Applying stringent criteria (|fold change| > 1.5 and adjusted *p*-value <0.05), we identified 700 differentially expressed proteins (DEPs). Venn diagram analysis showed that 684 proteins were altered in the model group compared to control, while CW treatment reversed 153 of these DEPs toward control levels (Figure 2B). Partial least squares-discriminant analysis (PLS-DA) of these DEPs revealed progressive separation among control, model, and CW_H groups, indicating therapeutic modulation of the hepatic proteome (Supplementary Figure S3).

KEGG pathway enrichment analysis of 43 core regulatory proteins revealed three interconnected biological networks significantly modulated by CW_H treatment (Figure 2C; Supplementary Figure S4): bile acid biosynthesis and cholesterol metabolism; NF-κB signaling and neutrophil extracellular trap (NET) formation; and cytochrome P450 mediated xenobiotic metabolism. These results suggest that CW acts on metabolic, inflammatory, and detoxification networks simultaneously.

### 3.3 Validation of bile acid metabolism and inflammatory pathway regulation

Based on proteomic insights, we performed targeted validation of key pathways using quantitative PCR and Western blot analyses. ANIT treatment significantly downregulated hepatic mRNA expression of bile acid synthesis enzymes (*Cyp7a1*, *Cyp27a1*, and *Cyp8b1*), reflecting feedback inhibition under cholestatic conditions. Notably, CW_H treatment significantly restored *Cyp27a1* expression and upregulated metabolizing enzymes (*Ugt1a1* and *Cyp3a11*) and bile acid efflux transporters (*Mrp2*, M*rp3,* and *Mrp4*), indicating enhanced hepatic detoxification capacity (Figures 2D–F).

Regarding inflammatory pathways, model mice exhibited significantly elevated mRNA levels of *Nf-κb*, *Tnf-α*, and *Il-1β*. CW treatment substantially suppressed these inflammatory mediators (Figure 2G). Western blot analysis confirmed significant downregulation of phosphorylated NF-κB (p-NF-κB), total NF-κB, IL-1β, and TNF-α at the protein level, validating the anti-inflammatory effects of CW (Figures 2H–K).

### 3.4 Structural characterization of CWP

To identify the bioactive constituents responsible for hepatoprotection, we performed systematic extraction and compositional analysis of *C. wenyujin*. Quantitative analysis showed that polysaccharides accounted for 9.45% (w/w) of the dried material, representing a major water-soluble constituent class and providing a rationale for evaluating their bioactivity.

**FIGURE 3:**
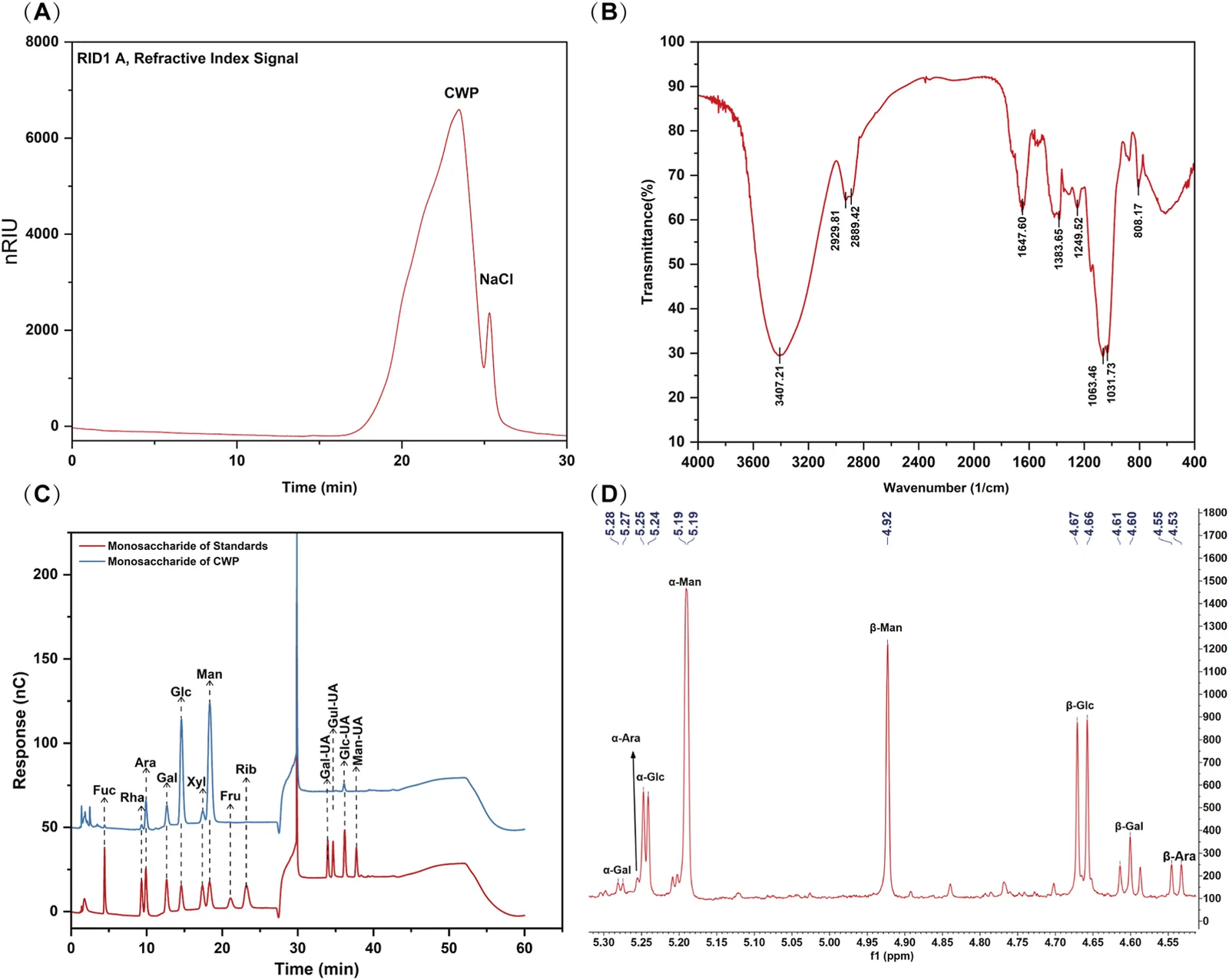
Structural characterization and monosaccharide composition analysis of CWP. **(A)** Molecular weight distribution profile of CWP determined by gel permeation chromatography. **(B)** Fourier-transform infrared (FT-IR) spectrum of CWP showing characteristic polysaccharide absorption peaks. **(C)** High-performance anion-exchange chromatography (HPAEC) profiles comparing the monosaccharide composition of mixed reference standards (red line) and CWP (blue line). **(D)** ^1^H-NMR spectrum of CWP recorded in D_2_O. Table 1 shows the detailed molar percentages of monosaccharides in CWP.

Following enzymatic starch removal (α-amylase and glucoamylase treatment) and dialysis purification, CWP was obtained with a yield of 12.3% (based on crude polysaccharide weight). Colorimetric analysis revealed 77.8% total carbohydrates, with less than 15.6% protein contamination. HPGPC analysis showed a relatively uniform molecular weight distribution with a dominant peak corresponding to an average molecular weight of 5.89 kDa (Figure 3A). Fourier-transform infrared (FT-IR) analysis showed typical polysaccharide characteristics (Figure 3B). A broad absorption band at 3407.21 cm^-1^ was attributed to O–H stretching vibrations, while peaks at 2929.81 cm^-1^ and 2889.42 cm^-1^ corresponded to C–H stretching of methyl and methylene groups. The absorption at 1647.60 cm^-1^ was assigned to O–H bending vibration associated with bound water. Peaks at 1383.65 cm^-1^ and 1249.52 cm^-1^ were related to C–H deformation and C–O stretching vibrations respectively. Characteristic peaks at 1063.46 cm^-1^ and 1031.73 cm^-1^ indicated the pyranose ring structures, while the peak at 808.17 cm^-1^ suggested the characteristic skeletal vibration of mannose residues (Li et al., 2025), confirming the polysaccharide nature of CWP. Monosaccharide composition analysis revealed that CWP consisted of fucose, arabinose, rhamnose, galactose, glucose, xylose, mannose and glucuronic acid in molar percentage of 0.4%, 6.32%, 1.16%, 5.12%, 33.67%, 4.56%, 47.05% and 1.73% respectively, with mannose as the predominant sugar residue (Figure 3C; Table 1). The ^1^H NMR spectrum of CWP exhibited eight anomeric proton signals at *δ*_H_ 5.28 (d, *J* = 3.8 Hz), 5.26 (br s), 5.25 (d, *J* = 3.8 Hz), 5.19 (br s), 4.92 (br s), 4.67 (d, *J* = 8.0 Hz), 4.61 (d, *J* = 8.0 Hz) and 4.55 (d, *J* = 7.8 Hz), corresponding to α-galactose, α-arabinose, α-glucose, α-mannose, β-mannose, β-glucose, β-galactose, and β-arabinose residues, respectively (Figure 3D).

**TABLE 1:** Monosaccharide compositions (mol %) of CWP.

Sample	Fucose	Arabinose	Rhamnose	Galactose	Glucose	Xylose	Mannose	Glucuronic acid
CWP	0.40%	6.32%	1.16%	5.12%	33.67%	4.56%	47.05%	1.73%

### 3.5 CWP pretreatment attenuates cholestatic liver injury in ANIT-challenged mice

**FIGURE 4:**
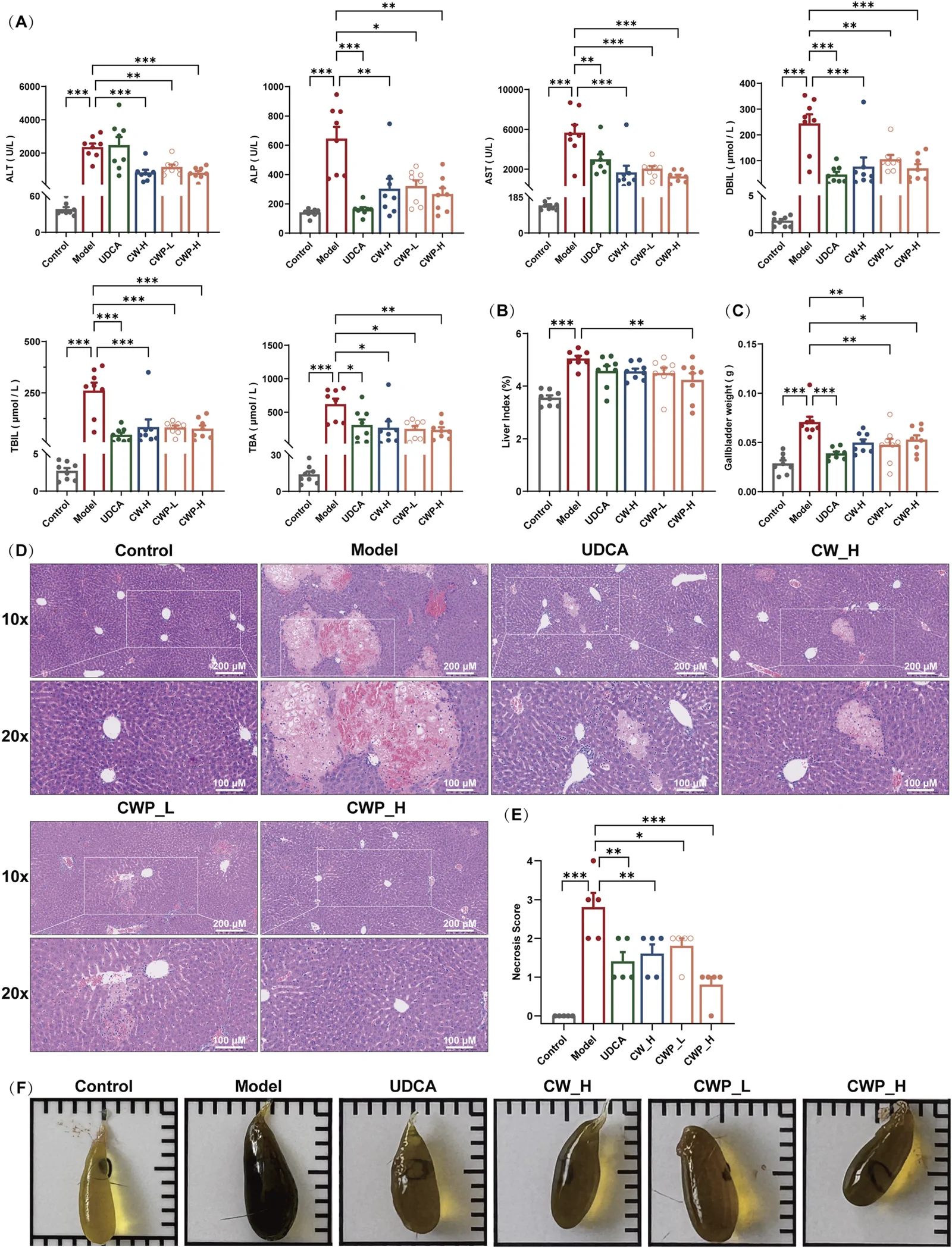
CWP attenuates ANIT-induced hepatobiliary dysfunction in mice. **(A)** Serum levels of hepatic injury markers (ALT, ALP, AST) and cholestasis indicators (DBIL, TBIL, TBA). **(B)** Liver index, calculated as the ratio of liver weight to body weight. **(C)** Quantitative evaluation of gallbladder weight. **(D)** Representative H&E-stained liver tissue sections evaluating hepatic necrosis and inflammatory infiltration (Magnification: ×10, scale bar = 200 μm; 20×, scale bar = 100 μm). **(E)** Histopathological necrosis score. **(F)** Gross morphological appearance of gallbladders, showing enlargement and dark discoloration in the Model group, which was alleviated by CWP treatment. Data are presented as mean ± SEM (n = 8 for serum biochemistry; n = 5 for necrosis score). Statistical significance was assessed by one-way ANOVA (*p < 0.05, **p < 0.01, ***p < 0.001 vs. Model group).

To investigate the contribution of polysaccharides to the overall efficacy of CW, we evaluated the hepatoprotective effects of purified CWP against ANIT-induced cholestatic liver injury in mice. CWP treatment at both doses significantly ameliorated biochemical markers of liver damage and cholestasis compared to the Model group, with numerically greater effects at the higher dose, the magnitude of improvement was qualitatively consistent with that observed in CW treated group (Figure 4A). Although CWP treatments did not significantly reverse the ANIT-induced body weight loss (Supplementary Figure S5), ANIT significantly elevated the liver index (liver to body weight ratio), whereas CWP administration effectively reversed this increase (Figure 4B). Histological examination revealed significant reductions in inflammatory cell infiltration and hepatocyte necrosis following CWP treatment (Figures 4D,E). Moreover, CWP intervention reduced gallbladder weight, normalized gallbladder morphology, and restored physiological bile coloration (Figures 4C,F), indicating that CWP, as a polysaccharide-enriched fraction, possesses significant hepatoprotective activity in this acute cholestasis model.

### 3.6 CWP selectively ameliorates gut microbiota composition in cholestatic mice

**FIGURE 5:**
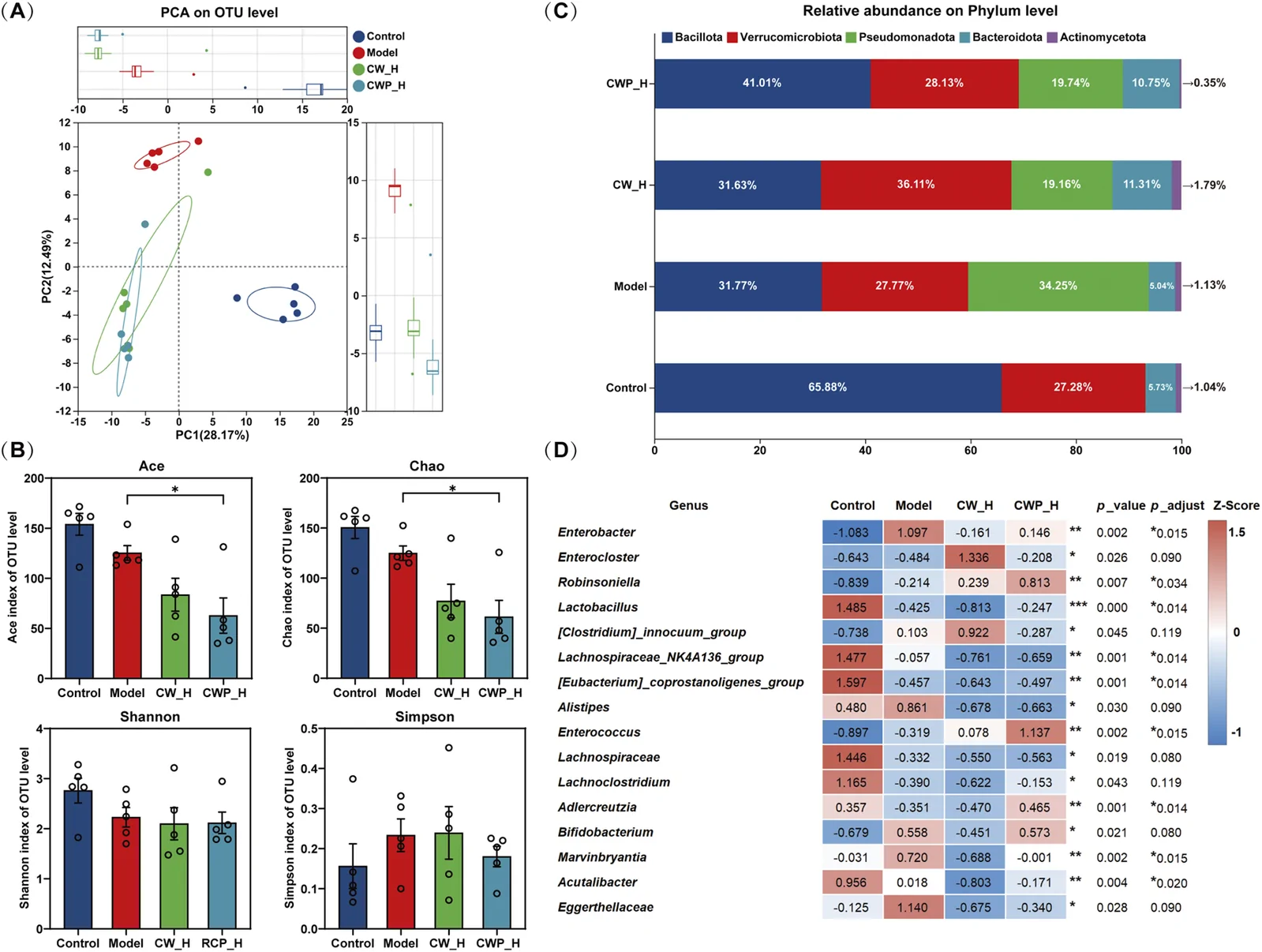
CWP modulate gut microbiota composition in ANIT-induced cholestatic mice. **(A)** PCA plot based on OTU-level abundance showing the distinct clustering of gut microbial communities among treatment groups. **(B)** Alpha-diversity indices including Ace, Chao, Shannon, and Simpson indices, reflecting microbial community richness and diversity. **(C)** Relative abundance of bacterial taxa at the phylum level. **(D)** Heatmap visualizing the Z-score normalized relative abundances of 16 significantly altered bacterial genera. *p*_value: Kruskal-Walli’s test (*p < 0.05, **p < 0.01, ***p < 0.001). *p*_adjust: Benjamini–Hochberg FDR correction (*p_adjust <0.05). Red and blue colors indicate higher and lower relative abundances, respectively. Data are presented as mean ± SEM (n = 5).

Given that proteomic analysis revealed significant enrichment of inflammatory pathways potentially linked to gut-derived signals, and considering the well-established role of the gut-liver axis in cholestatic pathogenesis, we investigated whether CWP’s hepatoprotective effects involve modulation of intestinal microbiota. Using 16S rRNA gene sequencing, PCA revealed distinct clustering patterns among experimental groups, with CW_H and CWP_H demonstrating similar microbiota profiles, suggesting comparable mechanisms of action (Figure 5A).

Alpha diversity analysis revealed a non-significant trend toward decreased Ace, Chao, and Shannon indices, alongside an increased Simpson index in the model group compared to controls, indicating mild dysbiosis induced by ANIT-mediated cholestatic injury. Interestingly, CWP_H treatment led to statistically significant further reductions in Ace and Chao indices relative to the model group, whereas Shannon and Simpson indices showed no significant changes (Figure 5B). This suggests that CWP_H selectively reduced species richness without markedly affecting microbial evenness or diversity. At the phylum level, model mice exhibited dramatic reductions in *Bacillota* (from 65.88% to 31.77%) and marked expansion of *Pseudomonadota* (from 0.06% to 34.25%) (Figure 5C). Both CW_H and CWP_H partially restored phylum-level balance by reducing *Pseudomonadota* and increasing *Bacteroidota* abundance.

Building on the alpha diversity and phylum-level findings, we performed Kruskal–Wallis test analysis on genus level bacterial abundance data, followed by Benjamini–Hochberg False Discovery Rate (FDR) correction. A total of 16 genera showed significant differences among groups (*p* < 0.05), 9 genera remained strictly significant among groups after FDR correction (*p* adjust <0.05). Their relative abundances were normalized by Z-score and visualized in a heatmap (Figure 5D; Supplementary Table S7). ANIT induced cholestatic liver injury markedly altered gut microbiota composition. Specifically, the abundances of *Enterobacter* were significantly increased in the Model group. In contrast, several bacteria were notably decreased, including *Lactobacillus*, *Lachnospiraceae_NK4A136_group*, and *Eubacterium_coprostanoligenes_group*. Both treatments modulated gut microbiota with distinct patterns. While CW_H showed limited regulation on these key genera and notably exhibited a selective expansion of *Enterocloster*, a genus reported to be associated with bile acid dysmetabolism, CWP_H demonstrated favorable selective regulation, characterized by partial restoration of *Lactobacillus*, full recovery of *Adlercreutzia*, normalization of *Marvinbryantia*, and suppression of the elevated *Enterobacter*. This differential microbiota modulating profile correlated with CWP_H’s superior therapeutic efficacy in biochemical and histopathological assessments.

Collectively, these findings indicate that CWP’s hepatoprotective effects are associated with selective remodeling of the gut microbial community, rather than complete restoration of baseline microbiota. This targeted modulation, characterized by suppression of pathobiont expansion and promotion of beneficial bacteria, likely contributes to rebalancing the gut-liver axis and ameliorating hepatic injury.

### 3.7 CWP treatment is associated with normalized bile acid homeostasis

Given the established role of intestinal microbiota in bile acid biotransformation through deconjugation and 7α-dehydroxylation, we investigated whether CWP mediated microbiota modulation translates into improved hepatic bile acid homeostasis. We first confirmed that CWP treatment recapitulated CW’s regulatory effects on hepatic gene expression profiles governing bile acid metabolism. Specifically, CWP maintained consistent upregulation of bile acid synthesis enzymes (*Cyp27a1* and *Cyp8b1*), efflux transporters (*Mrp2*, *Mrp3,* and *Mrp4*), and phase II detoxification enzymes (*Ugt1a1* and *Cyp3a11*), confirming mechanistic concordance between the purified polysaccharide and crude extract (Supplementary Figure S6). To assess whether these transcriptional changes translated into functional improvement in bile acid homeostasis, we performed targeted LC-MS/MS profiling of 26 bile acid species in liver tissues.

**FIGURE 6:**
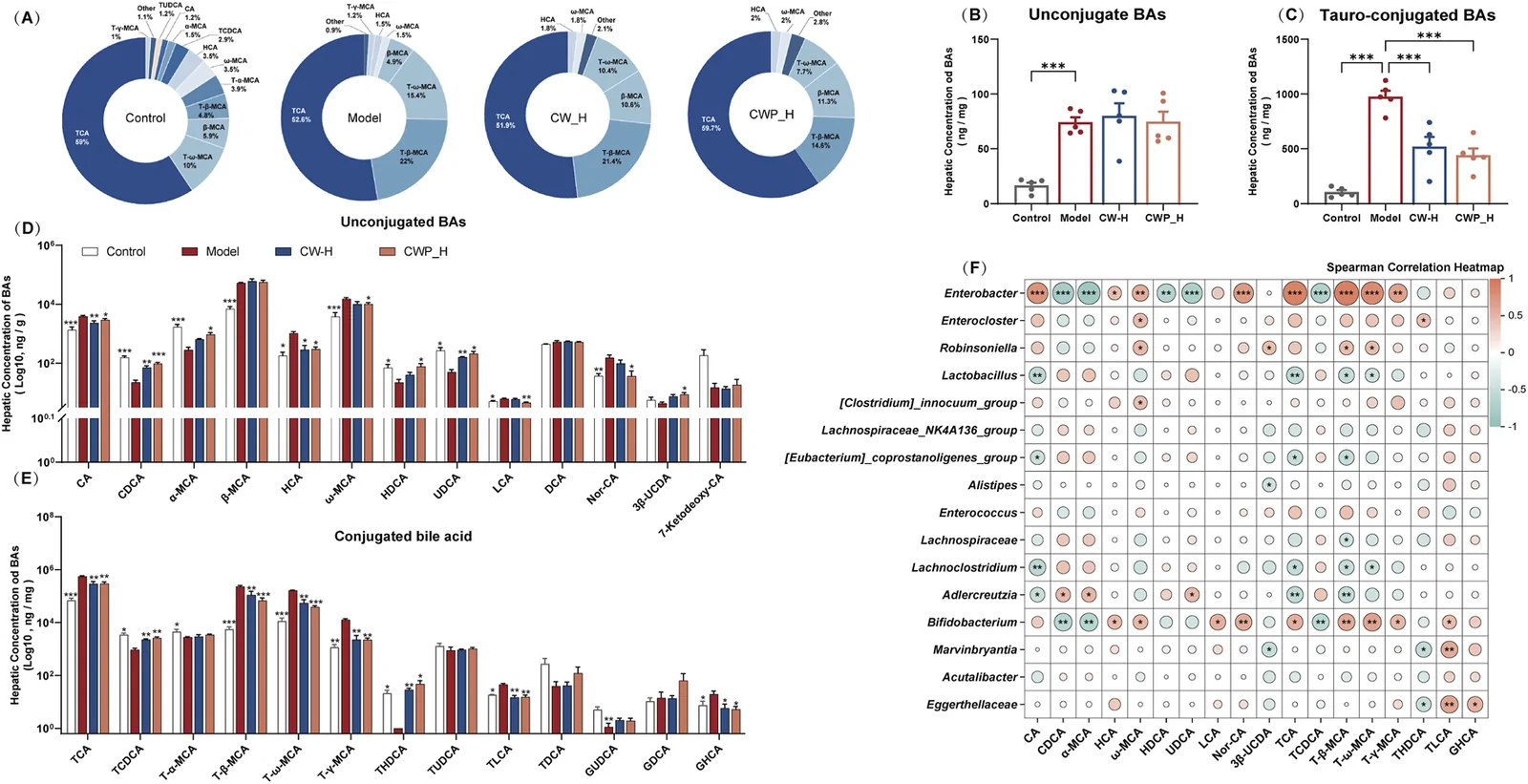
CWP restore hepatic bile acid homeostasis. **(A)** Pie charts illustrating the percentage distribution of major bile acid species in the liver across different groups. **(B,D)** Hepatic concentrations of total and individual unconjugated bile acids. **(C,E)** Hepatic concentrations of total and individual conjugated bile acids. **(F)** Spearman correlation heatmap illustrating associations between 16 differentially abundant bacterial genera (from Figure 5D) and hepatic bile acid species. Red indicates positive correlations, while blue indicates negative correlations (*p < 0.05, **p < 0.01, ***p < 0.001).

ANIT treatment induced severe disruption of hepatic bile acid homeostasis, characterized by significant accumulation of total bile acids with pronounced elevations in both conjugated and unconjugated forms. Notable compositional changes included pathological accumulation of Tauro-β-muricholic acid (T-β-MCA) (from 4.8% to 22.0% of total bile acids) and decreased proportions of major physiological conjugated bile acids, such as Taurocholic acid (TCA) and Taurochenodeoxycholic acid (TCDCA). CWP intervention effectively restored bile acid homeostasis, significantly reducing pathological T-β-MCA accumulation from 22.0% to 14.6% (*p* < 0.001) and T-ω-MCA levels from 15.4% to 7.7% (*p* < 0.001), while normalizing TCA proportions (from 52.6% to 59.7%) (Figure 6A). Mechanistically, CWP treatment reduced absolute concentrations of cytotoxic unconjugated and conjugated bile acids while specifically enhancing hepatoprotective species including UDCA and CDCA (Figures 6B–E). These improvements in bile acid profile CWP effectively modulates hepatic bile acid homeostasis at the tested dose.

To elucidate the mechanistic link between microbiota modulation and bile acid homeostasis, we performed Spearman correlation analysis between differentially abundant bacterial genera and hepatic bile acid profiles. Spearman correlation heatmap analysis revealed significant associations between specific bacterial taxa and bile acid species (Figure 6F). Notably, *Enterobacter,* which was markedly elevated in the model group and effectively suppressed by CWP_H treatment, exhibited strong positive correlations with cytotoxic bile acids including CA, TCA, T-β-MCA, Tauro-omega-muricholic acid (T-ω-MCA), and Tauro-gamma-muricholic acid (T-γ-MCA). Conversely, *Lactobacillus*, which was partially restored by CWP_H intervention, showed significant negative correlations with cytotoxic CA, TCA, T-β-MCA and T-ω-MCA. Similarly, the *Eubacterium_coprostanoligenes_group* demonstrated negative correlations with CA, TCA, and T-β-MCA. *Adlercreutzia*, which was fully restored by CWP_H, also negatively correlated with TCA and T-β-MCA. These correlation patterns suggest a potential link between CWP-mediated shifts in bacterial composition and the observed changes in hepatic bile acid profiles.

Collectively, these findings demonstrate that CWP actively regulates hepatic bile acid metabolism and simultaneously reshapes the gut microbiota. Furthermore, the strong correlations between specific microbial taxa and bile acid species suggest that an integrated modulation of the microbiota and bile acid axis is closely associated with the hepatoprotective effects of CWP.

### 3.8 CWP reduces hepatic neutrophil infiltration and restores intestinal barrier marker expression

**FIGURE 7:**
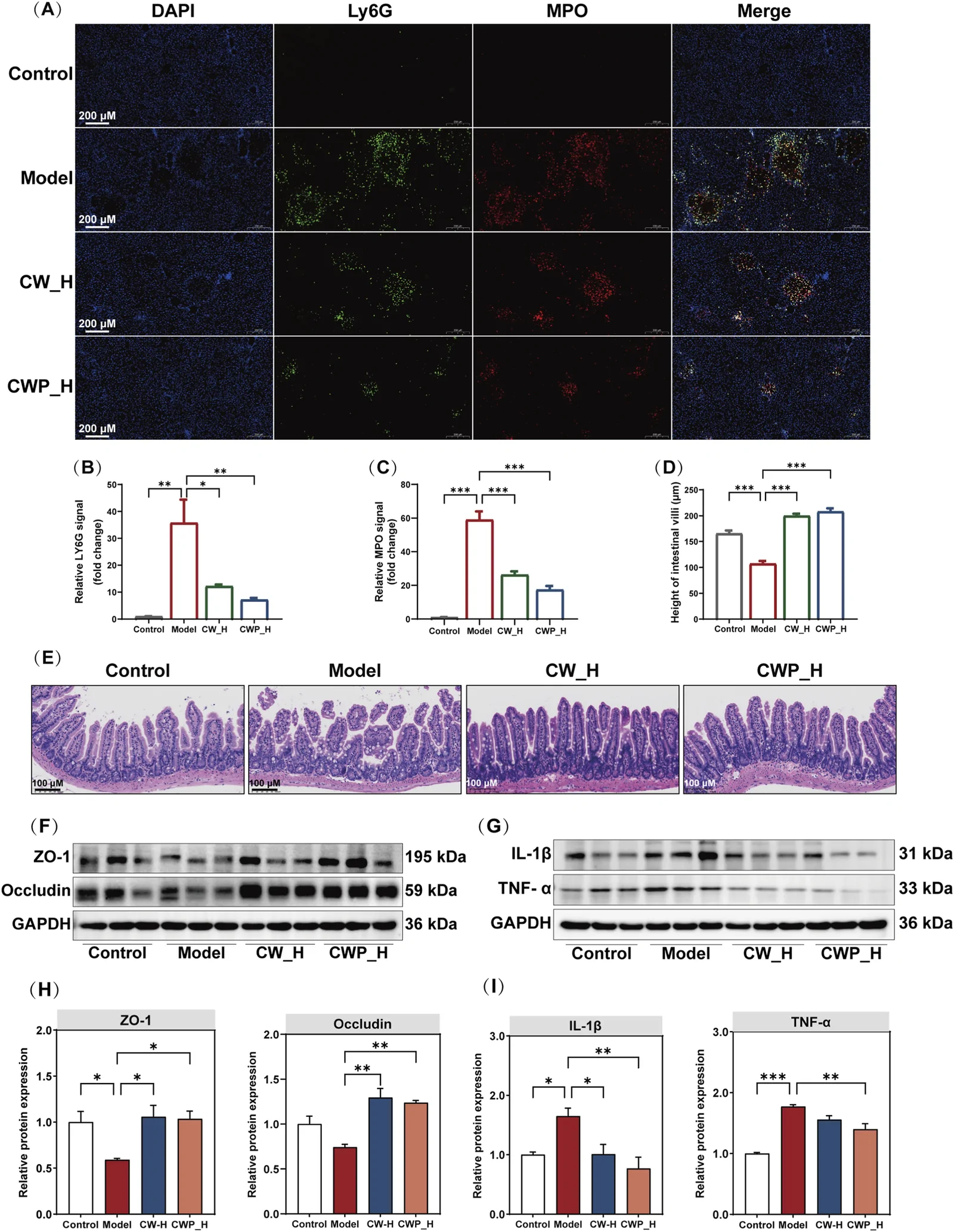
CWP reduces hepatic neutrophil infiltration and restores intestinal barrier marker expression. **(A)** Representative double immunofluorescence staining of liver sections for NET markers: Ly6G (green, neutrophils), MPO (red), and DAPI (blue, nuclei), magnification: ×10, scale bar = 200 μm. **(B,C)** Quantitative analysis of mean fluorescence intensity for Ly6G and MPO signals. **(D,E)** Representative H&E-stained ileum tissue sections showing intestinal morphological preservation (Magnification: ×20, scale bar = 100 μm). Quantitative analysis of the height of intestinal villi based on H&E staining. **(F,H)** Representative Western blot images and densitometric quantification of intestinal tight junction proteins (ZO-1 and Occludin) in colonic tissues. **(G,I)** Representative Western blot images and densitometric quantification of inflammatory cytokines (IL-1β and TNF-α) in colonic tissues. GAPDH served as the internal control. Data are presented as mean ± SEM. Statistical significance was determined by one-way ANOVA (*p < 0.05, **p < 0.01, ***p < 0.001 vs. model group).

Building upon proteomic findings indicating enrichment of inflammatory response pathways, including NET formation, we investigated CWP’s effects on hepatic neutrophil infiltration and intestinal barrier function. Immunofluorescence analysis of liver sections revealed intense Ly6G staining in ANIT-challenged mice, indicating marked neutrophil infiltration and elevated myeloperoxidase (MPO) expression. Both CW and CWP administration reduced Ly6G and MPO fluorescence intensity, with CWP showing a greater reduction at the tested doses, suggesting a strong suppressive effect on neutrophil mediated hepatic inflammation (Figures 7A–C).

Concurrently, bile acid-driven dysbiosis compromised intestinal epithelial barrier integrity. Histological evaluation of ileal sections by H&E staining revealed marked intestinal injury in the model group, characterized by shortened and sparsely arranged villi, epithelial shedding, and prominent inflammatory cell infiltration (Figures 7D,E). Both CW and CWP interventions alleviated these histopathological alterations, with improvements in villus morphology and reductions in inflammatory infiltration. Consistent with these histological changes, ileal tissues from cholestatic mice exhibited pronounced downregulation of the tight junction proteins ZO-1 and Occludin, accompanied by elevated expression of pro-inflammatory cytokines IL-1β and TNF-α. CWP intervention effectively restored ZO-1 and Occludin expression to near-physiological levels while suppressing IL-1β and TNF-α production (Figures 7F–I), indicating the restoration of mucosal barrier-associated proteins and reduction of local intestinal inflammatory responses.

## 4 Discussion

Cholestatic liver disease presents a complex clinical challenge driven by the intertwined pathologies of bile acid toxicity, persistent inflammation, and gut-liver axis dysfunction (Fuchs et al., 2025). This study demonstrates that polysaccharides isolated from *C*. *wenyujin* (CWP) protect against ANIT-induced cholestatic liver injury in mice. The evidence supports a mechanism involving simultaneous regulation of hepatic bile acid handling, intestinal microbiota composition, and inflammatory signaling across the gut-liver axis.

The most prominent feature of CWP’s efficacy is the restoration of hepatic bile acid homeostasis. During cholestasis, the liver mounts a compensatory response that includes suppressing bile acid synthesis and upregulating efflux routes to limit intracellular toxicity (Zollner et al., 2003). Our findings show that CWP does not simply reinforce one arm of this response; instead, it partially restores the expression of synthesis enzymes (*Cyp27a1*, *Cyp8b1*), phase II conjugation enzymes (*Ugt1a1*, *Cyp3a11*), and basolateral efflux transporters (*Mrp2*, *Mrp3*, *Mrp4*). The functional consequence shows a reduction in intrahepatic T-β-MCA (from 22.0% to 14.6%) and an increase in hepatoprotective species such as UDCA, suggesting that CWP promotes bile acid clearance by reactivating endogenous metabolic and excretory pathways rather than by acting as an exogenous bile acid supplement. This mechanistic profile differs fundamentally from current first-line therapies. UDCA primarily enriches the hydrophilic bile acid pool through direct incorporation, displacing cytotoxic hydrophobic species but without addressing the underlying metabolic dysfunction. Obeticholic acid (OCA), in contrast, activates FXR to suppress CYP7A1 mediated bile acid synthesis, effectively reducing the bile acid load but at the cost of dampening endogenous synthetic capacity and frequently provoking pruritus (Pellicciari et al., 2002). CWP’s strategy of simultaneously enhancing detoxification (phase II conjugation) and excretion (basolateral efflux) while preserving synthetic enzyme expression allows the liver to process and eliminate accumulated bile acids rather than merely blocking their production.

This hepatic metabolic shift is intricately linked to CWP’s regulatory effects on the intestinal environment. Cholestasis reduces bile acid delivery to the intestinal lumen, disrupting commensal communities that depend on bile acids as metabolic substrates (Ghallab et al., 2025; Kummen and Hov, 2019). We observed this as a marked expansion of *Enterobacter* and depletion of beneficial *Lactobacillus*, *Lachnospiraceae* members, and *Adlercreutzia* in the model group. CWP intervention selectively reversed key aspects of this dysbiosis. By suppressing *Enterobacter*, a taxon known to facilitate endotoxin translocation (Jiang et al., 2025; Zhang et al., 2022), and restoring *Lactobacillus* and *Adlercreutzia*, CWP is associated with a microbial environment that may be conducive to bile acid biotransformation. The restored taxa are well-documented producers of bile salt hydrolases (BSH) and short-chain fatty acids (Ni et al., 2023; Zhao et al., 2026), which aligns with the strong negative correlations we observed between *Lactobacillus* abundance and cytotoxic conjugated bile acids (TCA, T-β-MCA). Mechanistically, this connection forms a coherent pathway: bacterial BSH activity deconjugates taurine/glycine-conjugated bile acids in the intestinal lumen, generating unconjugated species that can either be reabsorbed for hepatic reprocessing or further transformed into secondary bile acids by 7α-dehydroxylating bacteria (Begley et al., 2005). Based on the known biological roles of these bacteria, the CWP-associated enrichment of BSH-producing taxa may facilitate the deconjugation of accumulated T-β-MCA and TCA, thereby potentially reducing their hepatic burden and expanding the substrate pool for hepatoprotective species such as UDCA. This interpretation aligns with our concurrent observations of decreased hepatic T-β-MCA proportions and elevated UDCA levels following CWP treatment, providing a plausible mechanistic framework that links the parallel changes in microbiota composition and bile acid profiles.

The concurrent restoration of microbiota and bile acid balance ultimately translates into profound anti-inflammatory benefits across the gut-liver axis. In the gut, CWP restores the expression of epithelial tight junctions (ZO-1, Occludin), which is critically associated with the maintenance of intestinal barrier integrity. Consequently, we observed a dramatic attenuation of hepatic inflammatory signaling, highlighted by reduced NF-κB phosphorylation and downstream cytokine (TNF-α, IL-1β) production. More importantly, CWP effectively minimized hepatic Ly6G neutrophil infiltration and MPO expression. This finding is particularly relevant given recent evidence that neutrophil extracellular traps (NETs) act as key drivers of sterile inflammation and tissue damage in cholestatic conditions (Hintermann et al., 2024; Gujral et al., 2003), suggesting that reducing neutrophil infiltration and associated inflammatory activity contributes to CWP’s hepatoprotective effect. Collectively, these findings position CWP as a multi-target intervention that interrupts the self-amplifying cycle of bile acid toxicity, barrier dysfunction, and inflammatory amplification that characterizes progressive cholestatic injury.

The CW dosage was established based on the clinical usage recommendations in Chinese Pharmacopoeia, which recommends a daily dose of 3–10 g/day for *C. wenyujin* in adults. Applying the standard human to mouse body surface area conversion factor (approximately 9.1), the mouse equivalent dose is approximately 1.43 g raw material/kg. Accordingly, we set the CW doses at 0.75 and 1.5 g/kg (raw material equivalent). However, the dosage of CWP was not determined according to its proportional content in the aqueous extract, but rather selected as an independent pharmacological intervention dose based on commonly reported therapeutic range of 50–200 mg/kg for plant-derived polysaccharides in rodent models of liver disease (Xiao et al., 2025; Nie et al., 2025; Ling et al., 2026).

CWP was obtained as a crude polysaccharide fraction with 77.8% total carbohydrate content and less than 15.6% protein. The remaining constituents (∼6.6%) likely include lignin, inorganic salts, fat and bound phenolics commonly found in plant polysaccharide preparations at this purification stage (Shi, 2016; Ren et al., 2019). The notable protein content deserves brief consideration. In many plant-derived polysaccharide preparations, residual proteins can exist either as co-extracted contaminants or as components of naturally occurring glycoprotein complexes (Nie et al., 2025; Xue et al., 2022). Whether the protein fraction in CWP forms a functional glycoprotein assembly that contributes to the observed pharmacological effects, or simply represents a residual impurity, cannot be determined from the present data. Future studies involving protease digestion followed by bioactivity reassessment would help clarify this point.

CWP is a mannose and glucose rich polysaccharide-enriched fraction with an average molecular weight of 5.89 kDa. Polysaccharides with similar monosaccharide compositions from other plant sources, including glucomannans from *Dendrobium officinale* and mannose rich fractions from *Bletilla striata*, *have* demonstrated prebiotic and immunomodulatory properties (Wan et al., 2026; Pang et al., 2026). This low molecular weight may facilitate its ready fermentation by colonic bacteria, thereby driving the observed compositional shifts in the microbiome and supporting intestinal barrier integrity. However, since CWP was not further fractionated by ion exchange chromatography, it likely represents a mixture of polysaccharide components rather than a single homogeneous species.

Despite these encouraging findings, several limitations must be acknowledged. First and most importantly, while we demonstrated strong correlations between gut microbiota changes and hepatoprotective outcomes, the current study does not establish a direct causal relationship between microbial remodeling and bile acid normalization. Demonstrating that microbial remodeling is a necessary condition for CWP’s efficacy would require additional experimental validation, such as fecal microbiota transplantation, antibiotic mediated microbiota depletion prior to CWP treatment, or gnotobiotic mouse experiments. These experiments are planned for future investigation. Second, the detailed fine structure of CWP including specific linkage positions and branching patterns awaits further elucidation following chromatographic fractionation and 2D NMR analysis. Third, the contribution of the residual protein fraction in CWP to the observed bioactivity has not been addressed and merits future clarification. Finally, the ANIT model represents an acute cholestatic injury, and the long-term safety and efficacy of CWP in chronic or progressive fibrotic cholestasis models remain important topics for future study.

## 5 Conclusion

**FIGURE 8:**
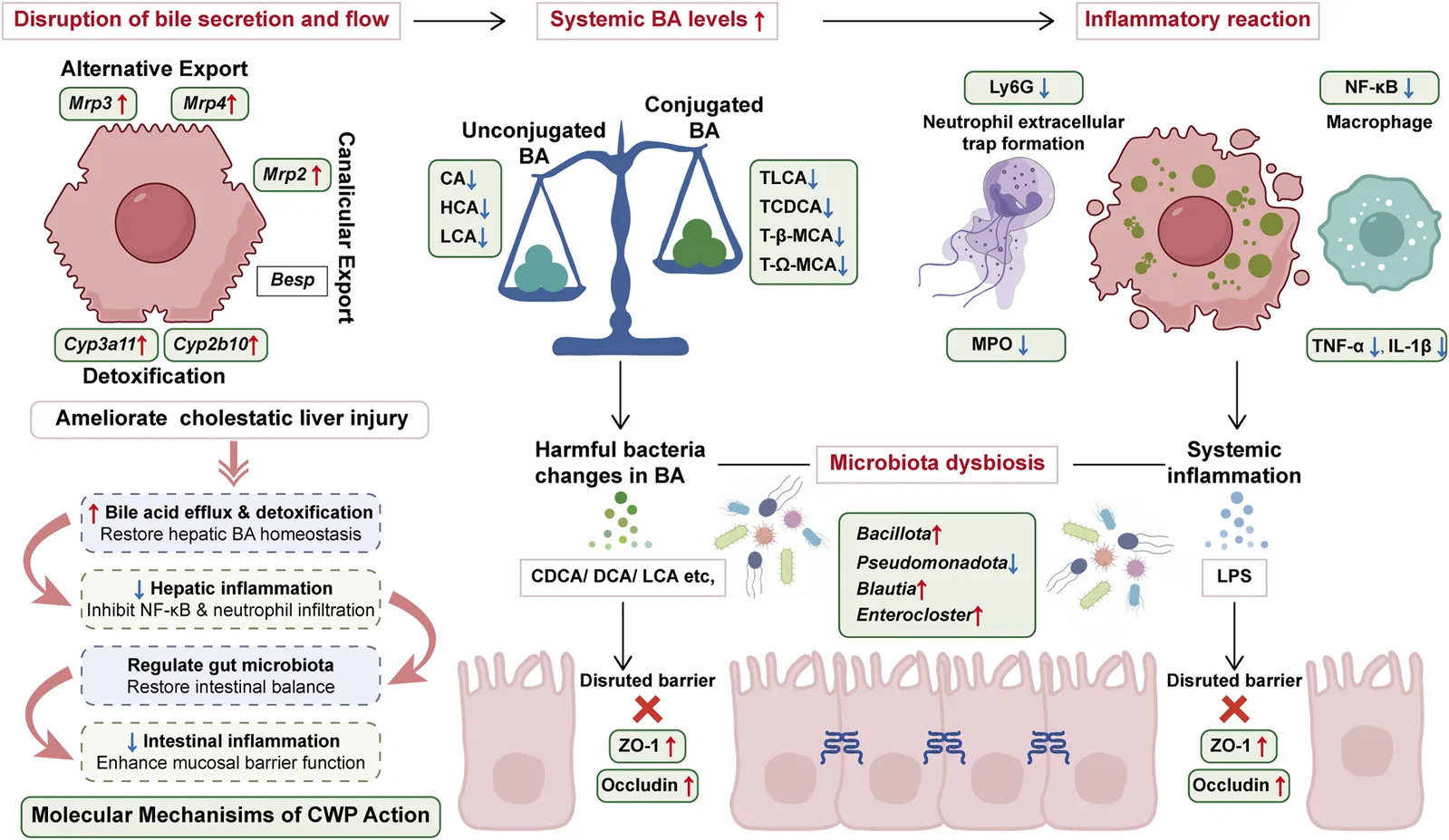
Proposed mechanism of *Curcuma wenyujin* polysaccharides in protecting against ANIT-induced cholestasis. In the liver, CWP mitigates intracellular bile acid (BA) overload by upregulating BA detoxification enzymes (*Cyp3a11*, *Cyp2b10*) and efflux transporters (*Mrp2*, *Mrp3*, *Mrp4*). This promotes BA clearance and restores the composition of the BA pool, significantly reducing the proportion of toxic conjugated BAs (e.g., TLCA, T-β-MCA). Furthermore, CWP exerts anti-inflammatory effects by inhibiting macrophage-mediated NF-κB signaling (downregulating TNF-α and IL-1β) and actively suppressing neutrophil infiltration (evidenced by decreased Ly6G and MPO levels). In the intestine, CWP ameliorates microbiota dysbiosis by modulating the abundance of specific bacterial taxa (e.g., increasing *Bacillota* and *Blautia*, while decreasing *Pseudomonadota*). This microbial reshaping, combined with the restored expression of tight junction proteins (ZO-1 and Occludin), repairs the intestinal mucosal barrier, thereby preventing the systemic translocation of harmful metabolites and lipopolysaccharide (LPS). Red arrows indicate upregulation or promotion by CWP treatment, while blue arrows denote downregulation or inhibition. Red arrows indicate upregulation by CWP, while blue arrows denote downregulation.

This study demonstrates that CWP pretreatment protects against acute ANIT-induced cholestatic liver injury in mice, operating at the intersection of host metabolism and microbial ecology (summarized in Figure 8). By selectively remodeling the gut microbiota, preserving intestinal barrier integrity, and reactivating hepatic bile acid clearance pathways, CWP pretreatment attenuates the pathological feedback loop of bile acid toxicity and inflammation in this acute model. These mechanistic insights not only validate the traditional application of *C*. *wenyujin* in biliary disorders but also highlight the therapeutic potential of targeting the gut-liver axis with plant-derived polysaccharides. Future work will focus on establishing causal relationships through microbiota intervention experiments and elucidating the complete structure of CWP to enable precise structure-activity analysis.

## Data Availability

The original contributions presented in the study are publicly available. The mass spectrometry proteomics data can be found in the ProteomeXchange repository (dataset identifier: PXD081104; https://proteomecentral.proteomexchange.org/cgi/GetDataset?ID=PXD081104). The gut microbiota 16S rRNA sequencing data can be found in the NCBI Sequence Read Archive (SRA) repository (Accession number: PRJNA1469391; https://www.ncbi.nlm.nih.gov/sra/PRJNA1469391).
