# Supplementary material for: Polysaccharides from the root tubers of Curcuma wenyujin ameliorate cholestatic liver injury associated with remodeling gut microbiota and restoring bile acid homeostasis
Source: Front Pharmacol. 2026 Sep 3;17:1847544. doi: 10.3389/fphar.2026.1847544 (PMC13582482; doi:10.3389/fphar.2026.1847544)
Supplement: Supplementary file 1 [file DataSheet1.docx]

**Supporting Information**

**Polysaccharides from the root tubers of *Curcuma wenyujin* ameliorate cholestatic liver injury associated with remodeling gut microbiota and restoring bile acid homeostasis**

Juan Zou^1^, Yuxin Kan^1^, Wenqi Song^1^, Shuyu Song^1^, Doudou Huang^1^, Jingwen Liu^2*^, Fujiang Guo^1,3*^

^1^ School of Pharmacy, Shanghai University of Traditional Chinese Medicine, Shanghai, 201203, P. R. China

^2^ Experiment Center for Science and Technology, Shanghai University of Traditional Chinese Medicine, Shanghai, 201203, P. R. China

^3^ Shanghai Seventh People's Hospital, Shanghai University of Traditional Chinese Medicine, Shanghai 200137, P. R. China

*** Correspondence:**
Corresponding Author
ljw540558623@shutcm.edu.cn；

gfj@shutcm.edu.cn

| **Contents** | **Page** |
| --- | --- |
| **Supplementary methods** | S1 |
| **Figure S1.** HPLC-DAD chemical profile of eight characteristic sesquiterpenoids in the *C. wenyujin* | S2 |
| **Figure S2.** Summary of identified and quantified proteins. | S3 |
| **Figure S3.** Partial least squares-discriminant analysis (PLS-DA) of proteomic profiles across experimental groups | S3 |
| **Figure S4.** Heatmap of the 43 core proteins selected by VIP score > 1.5 across all experimental groups. | S4 |
| **Figure S5.** Body weight changes of mice in Experiment I and Experiment II. | S5 |
| **Figure S6.** CWP maintains regulatory effects of crude extract on bile acid-related and inflammation genes expression | S6 |
| **Table S1.** The calibration curves, correlation coefficients (R2), and linear ranges of eight characteristic sesquiterpenoids in the *C. wenyujin* | S7 |
| **Table S2.** Contents of the eight characteristic sesquiterpenoids in the *C. wenyujin* | S7 |
| **Table S3.** Primer sequences | S8 |
| **Table S4.** Standard curve and regression coefficient of monosaccharide standards. | S8 |
| **Table S5.** Selection of 43 Core Proteins by VIP Score > 1.5. | S9-S10 |
| **Table S6.** Calibration curve data and QC RSDs of 26 liver bile acids | S10 |
| **Table S7.** Relative abundance of key differential gut microbiota genera across experimental groups. | S11 |

**Supplementary methods**

**HPLC analysis of sesquiterpenoid metabolites in *C. wenyujin***

Sample preparation: Powdered root tubers 25 mg were accurately weighed and ultrasonically extracted with methanol 1 mL for 45 min at room temperature. The extract was centrifuged at 10000 rpm for 10 min, and the supernatant was filtered through a 0.22 μm membrane prior to analysis.

Standard preparation: 13-hydroxygermacrone, (4S,5S) -(+)-germacrone-4,5-epoxide, curcumenone, dehydrocurdione, neocurdione, curdione, germacrone, and furanodiene were accurately weighed and dissolved in methanol to prepare individual stock solutions (2–5 mg/mL). Appropriate volumes of each stock solution were mixed and serially diluted with methanol to yield working standard solutions at various concentrations for calibration.

Chromatographic conditions: HPLC analysis was performed on an Agilent 1260 Infinity II LC system equipped with a DAD detector. Chromatographic separation was achieved on an Eclipse XDB-C18 (4.6×250 mm, 5 μm) maintained at 35 °C. The mobile phase consisted of water (A) and acetonitrile (B), eluted at a flow rate of 1.0 mL/min with the following gradient program: 0-13 min：48%B，13-15 min：48%-57%B，15-23 min：57%-61%B，23-26 min：61%-65%B，26-27 min：65%-70%B，27-30 min：70%B，30-32 min：70%-93%B，32-40 min：93%B，40-40.1 min：93%-48%B，40.1-43 min: 48%B. The injection volume was 10 μL, and the detection wavelength was set at 230 nm.

**Supplementary figures**

**Figure S1.** HPLC-DAD chemical profile of eight characteristic sesquiterpenoids in the *C. wenyujin*. Representative chromatograms were recorded at 230 nm. (A) The mixed reference standards. (B) *C. wenyujin* root tubers. The annotated peaks correspond to the following identified metabolites: 13-hydroxygermacrone, (4S,5S) -(+)-germacrone-4,5-epoxide, curcumenone, dehydrocurdione, neocurdione, curdione, germacrone, and furanodiene.

**Figure S2.** Summary of identified and quantified proteins.

**Figure S3.** Partial least squares-discriminant analysis (PLS-DA) of proteomic profiles across experimental groups.

**Figure S4.** Heatmap of the 43 core proteins selected by VIP score > 1.5 across all experimental groups.

**Figure S5.** Body weight changes of mice in Experiment I (A) and Experiment II (B). Data are presented as mean ± SEM (n = 8). Statistical significance was assessed by two-way ANOVA (**p* < 0.05 vs. model group).

**Figure S6.** CWP maintains regulatory effects of crude extract on bile acid-related and inflammation genes expression. Data are presented as mean ± SEM (n = 5). Statistical significance was assessed by one-way ANOVA (**p* < 0.05, ***p* < 0.01, ****p* < 0.001 vs. model group).

**Supplementary tables**

**Table S1. The calibration curves, correlation coefficients (R^2^), and linear ranges of eight** **characteristic sesquiterpenoids in the *C. wenyujin***

| **Compounds** | **Calibration curve** | **R^2^** | **Linear range**  **(μg/mL)** | **Retention time (min)** |
| --- | --- | --- | --- | --- |
| 13-hydroxygermacrone | Y=0.1114x+0.0949 | 0.9997 | 0.1-100 | 9.28 |
| (4S,5S) -(+)-germacrone-4,5-epoxide | Y=0.1681x+0.5941 | 0.9997 | 0.65-646 | 12.28 |
| curcumenone | Y=0.1681x+0.6601 | 0.9997 | 0.85-853 | 12.95 |
| dehydrocurdione | Y=0.1688x+0.3692 | 0.9997 | 0.41-410 | 14.09 |
| neocurdione | Y=0.2126x+0.3258 | 0.9997 | 0.5-500 | 17.69 |
| curdione | Y=0.1747x+1.3430 | 0.9997 | 2-2000 | 19.30 |
| germacrone | Y=0.1048x+0.1342 | 0.9997 | 0.6-600 | 31.90 |
| furanodiene | Y=0.1223x-3.5315 | 0.9993 | 2-2000 | 37.25 |

**Table S2. Contents of the eight characteristic sesquiterpenoids in the *C. wenyujin***

| Content  (mg/ g *C. wenyujin*) | Batch no. 20230223 | | | Batch no. 20240322 | | | Batch no. 20241202 | | |
| --- | --- | --- | --- | --- | --- | --- | --- | --- | --- |
|  | Mean |  | SD | Mean |  | SD | Mean |  | SD |
| 13-hydroxygermacrone | 6.015 | ± | 0.298 | 10.402 | ± | 0.667 | 4.205 | ± | 0.205 |
| (4S,5S)-(+)-germacrone-4,5-epoxide | 22.519 | ± | 1.624 | 26.027 | ± | 2.070 | 36.874 | ± | 1.924 |
| curcumenone | 111.317 | ± | 6.269 | 206.860 | ± | 13.033 | 84.878 | ± | 4.350 |
| dehydrocurdione | 9.801 | ± | 0.295 | 2.578 | ± | 0.136 | 6.097 | ± | 0.532 |
| neocurdione | 52.393 | ± | 6.811 | 51.291 | ± | 4.488 | 41.514 | ± | 9.874 |
| curdione | 216.060 | ± | 11.410 | 275.862 | ± | 17.207 | 221.848 | ± | 11.021 |
| germacrone | 45.700 | ± | 2.404 | 74.188 | ± | 4.674 | 81.278 | ± | 4.047 |
| furanodiene | 66.516 | ± | 3.549 | 45.287 | ± | 3.882 | 59.097 | ± | 3.570 |

**Table S3. Primer sequences**

| **Gene name** | **Primer sequence (5’ - 3’)** | |
| --- | --- | --- |
|  | **Forward** | **Reverse** |
| Cyp7a1 | GGGATTGCTGTGGTAGTGAGC | GGTATGGAATCAACCCGTTGTC |
| Cyp27a1 | CCAGGCACAGGAGAGTACG | GGGCAAGTGCAGCACATAG |
| Cyp8b1 | CCTCTGGACAAGGGTTTTGTG | GCACCGTGAAGACATCCCC |
| Ugt1a1 | GCTTCTTCCGTACCTTCTGTTG | GCTGCTGAATAACTCCAAGCAT |
| Cyp2b10 | AAAGTCCCGTGGCAACTTCC | TTGGCTCAACGACAGCAACT |
| Cyp3a11 | GGATGAGATCGATGAGGCTCTG | CAGGTATTCCATCTCCATCACAGT |
| Mrp2 | GTGTGGATTCCCTTGGGCTTT | CACAACGAACACCTGCTTGG |
| Mrp3 | CTGGGTCCCCTGCATCTAC | GCCGTCTTGAGCCTGGATAAC |
| Mrp4 | AGGAGCTTCAACGGTACTGG | GCCTTTGTTAAGGAGGGCTTC |
| Nf-κb | ATGGCAGACGATGATCCCTAC | TGTTGACAGTGGTATTTCTGGTG |
| Tnf-α | CCCTCACACTCAGATCATCTTCT | GCTACGACGTGGGCTACAG |
| Il-1β | TCCAGGATGAGGACATGAGCAC | GAACGTCACACACCAGCAGGTTA |
| Gapdh | AGGTCGGTGTGAACGGATTTG | TGTAGACCATGTAGTTGAGGTCA |

**Table S4. Standard curve and regression coefficient of monosaccharide standards.**

| **Monosaccharide** | **Abbreviation** | **Retention time**  **（min）** | **Regression equation** | **R^2^** |
| --- | --- | --- | --- | --- |
| Fucose | Fuc | 4.39 | Y = 1.1559 x + 0.7128 | 0.9958 |
| Rhamnose | Rha | 9.30 | Y = 1.3057 x + 0.4701 | 0.9983 |
| Arabinose | Ara | 9.87 | Y = 1.6532 x + 1.2023 | 0.9938 |
| Galactose | Gal | 12.60 | Y = 1.5939 x + 0.7696 | 0.9982 |
| Glucose | Glc | 14.54 | Y = 1.4651 x + 0.3862 | 0.9995 |
| Xylose | Xyl | 17.33 | Y = 1.4870 x + 0.5561 | 0.9994 |
| Mannose | Man | 18.26 | Y = 1.5062 x + 0.8073 | 0.9987 |
| Fructose | Fru | 21.01 | Y = 0.5546 x + 0.1141 | 0.9999 |
| Ribose | Rib | 23.08 | Y = 1.3195 x + 0.6280 | 0.9990 |
| Galacturonic Acid | Gal-UA | 34.02 | Y = 0.6050 x + 0.5187 | 0.9984 |
| Guluronic Acid | Gul-UA | 34.72 | Y = 0.6160 x + 0.5061 | 0.9978 |
| Glucuronic Acid | Glc-UA | 36.25 | Y = 1.0402 x + 0.4332 | 0.9998 |
| Mannuronic Acid | Man-UA | 37.79 | Y = 0.6420 x + 0.2846 | 0.9997 |

**Table S5. Selection of 43 Core Proteins by VIP Score > 1.5.**

| **NO.** | **Protein_ID** | **VIP_score** | **Gene_Name** |
| --- | --- | --- | --- |
| 1 | P35803 | 2.490 | Gpm6b |
| 2 | Q9DCG2 | 2.483 | Cd302 |
| 3 | Q08481 | 2.393 | Pecam1 |
| 4 | Q921I2 | 2.176 | Klhdc4 |
| 5 | Q8R151 | 2.117 | Znfx1 |
| 6 | Q8R323 | 2.093 | Rfc3 |
| 7 | P15392 | 2.070 | Cyp2a4 |
| 8 | P47811 | 2.053 | Mapk14 |
| 9 | O88824 | 2.043 | Jtb |
| 10 | Q05816 | 2.041 | Fabp5 |
| 11 | Q3TBD2 | 2.002 | Arhgap45 |
| 12 | Q3UHQ6 | 1.991 | Dop1b |
| 13 | Q3TYS2 | 1.978 | Cybc1 |
| 14 | P02104 | 1.971 | Hbb-y |
| 15 | Q91Y74 | 1.954 | St3gal4 |
| 16 | O35680 | 1.953 | Mrps12 |
| 17 | Q920S3 | 1.937 | Gatad1 |
| 18 | Q91XC8 | 1.914 | Dap |
| 19 | A2APU8 | 1.904 | *NA* |
| 20 | Q8R1H0 | 1.899 | Hopx |
| 21 | G3X8Q5 | 1.875 | Cp |
| 22 | Q9JIX0 | 1.874 | Eny2 |
| 23 | A0A0N4SW65 | 1.873 | Cmas |
| 24 | P97473 | 1.849 | Tarbp2 |
| 25 | Q6Y7W8 | 1.811 | Gigyf2 |
| 26 | P41245 | 1.760 | Mmp9 |
| 27 | P24721 | 1.759 | Asgr2 |
| 28 | G3X9W0 | 1.738 | Hnrnpd |
| 29 | P68433 | 1.734 | H3c1 |
| 30 | Q62203 | 1.709 | Sf3a2 |
| 31 | Q8K5B2 | 1.695 | Mcfd2 |
| 32 | P43883 | 1.671 | Plin2 |
| 33 | Q91W64 | 1.638 | Cyp2c70 |
| 34 | P56383 | 1.631 | Atp5mc2 |
| 35 | Q9R008 | 1.627 | Mvk |
| 36 | Q9CYA0 | 1.623 | Creld2 |
| 37 | Q9D110 | 1.622 | Mthfs |
| 38 | P21570 | 1.618 | Ang |
| 39 | P56135 | 1.594 | Atp5mf |
| 40 | Q8K354 | 1.577 | Cbr3 |
| 41 | Q61153 | 1.543 | Dio1 |
| 42 | E9QA16 | 1.535 | Cald1 |
| 43 | Q9D4V7 | 1.534 | Rabl3 |

**Table S6. Calibration Curve Data and QC RSDs of 26 Liver Bile Acids**

| **Bile acid** | **Calibration curve** | **R^2^** | **Liner range (μg/mL)** | **QC stability RSD （%）** |
| --- | --- | --- | --- | --- |
| CA | Y = 0.01719 x + 0.00359 | 0.998 | 0.2-1000 | 4.01 |
| CDCA | Y = 0.01045 x + 0.00141 | 0.9984 | 0.2-1000 | 2.54 |
| α-MCA | y = 0.00478 x + 4.40662e^-4^ | 0.9995 | 0.2-1000 | 2.95 |
| β-MCA | Y = 0.00149 x + 6.28576e-^4^ | 0.9992 | 1-1000 | 9.21 |
| HCA | Y = 0.00879 x - 3.63280e^-4^ | 0.9991 | 0.2-200 | 1.28 |
| ω-MCA | Y = 0.00244 x - 5.95412e^-4^ | 0.9986 | 1-1000 | 1.21 |
| HDCA | Y = 0.00528 x - 2.89257e^-4^ | 0.9988 | 0.2-1000 | 1.51 |
| UDCA | Y = 0.01268 x + 3.07400e^-4^ | 0.9985 | 0.2-1000 | 4.08 |
| LCA | Y = 0.01755 x - 0.00141 | 0.9994 | 0.5-1000 | 2.79 |
| DCA | Y = 0.00664 x + 0.03674 | 0.9995 | 5-1000 | 2.45 |
| Nor-CA | y = 0.00689 x + 7.51530e^-4^ | 0.9983 | 0.2-1000 | 2.11 |
| 3 β-UDCA | Y = 0.01148 x + 0.00135 | 0.9987 | 0.2-1000 | 1.44 |
| 7-ketodeoxy-CA | Y = 0.01032 x + 7.45000e^-4^ | 0.9985 | 0.2-1000 | 2.23 |
| TCA | Y = 0.00794 x + 9.29397e^-4^ | 0.9982 | 0.2-1000 | 3.37 |
| TCDCA | Y = 0.02053 x + 0.00209 | 0.9993 | 0.2-1000 | 3.39 |
| T-α-MCA | Y = 0.03869 x + 0.00255 | 0.9993 | 0.2-1000 | 1.25 |
| T-β-MCA | Y = 0.04218 x + 0.00226 | 0.9989 | 0.2-1000 | 2.77 |
| T-ω-MCA | Y = 0.04392 x + 0.00231 | 0.9991 | 0.2-1000 | 1.67 |
| T-γ-MCA/THCA | Y = 0.04085 x + 4.95908e^-5^ | 0.9983 | 0.2-1000 | 1.67 |
| THDCA | Y = 0.07498 x + 0.00458 | 0.9992 | 0.2-1000 | 2.95 |
| TUDCA | Y = 0.02175 x + 0.00128 | 0.9985 | 0.2-1000 | 3.92 |
| TLCA | Y = 0.03038 x + 0.00263 | 0.9994 | 0.2-1000 | 1.01 |
| TDCA | Y = 0.02310 x + 0.00185 | 0.9987 | 0.2-1000 | 2.94 |
| GUDCA | Y = 0.03275 x - 0.00143 | 0.9988 | 0.2-1000 | 0.34 |
| GDCA | Y = 0.04543 x - 5.50529e^-4^ | 0.9995 | 0.2-1000 | 0.99 |
| GHCA | Y = 0.01645 x + 0.00123 | 0.9981 | 0.2-200 | 1.93 |

**Table S7. Relative abundance of key differential gut microbiota genera across experimental groups.**

| **Species name** | **Control-Mean (%)** | **Control-Sd (%)** | **Model-Mean (%)** | **Model- Sd (%)** | **CW_H-Mean (%)** | **CW_H-Sd (%)** | **CWP_H-Mean (%)** | **CWP-Sd (%)** | ***p*_value** | ***p*_adjust** |
| --- | --- | --- | --- | --- | --- | --- | --- | --- | --- | --- |
| g__Enterobacter | 0.05192 | 0.04596 | 33.33 | 13.35 | 14.12 | 9.545 | 18.81 | 11.46 | 0.0022 | 0.01479 |
| g__Enterocloster | 0.07528 | 0.04797 | 1.504 | 1.698 | 17.91 | 10.34 | 3.992 | 4.979 | 0.02616 | 0.08968 |
| g__Robinsoniella | 0.00156 | 0.00348 | 3.31 | 5.707 | 5.703 | 2.533 | 8.738 | 6.517 | 0.00721 | 0.03366 |
| g__Lactobacillus | 10.41 | 2.536 | 1.774 | 2.556 | 0.01402 | 0.01369 | 2.577 | 2.182 | 0.00047 | 0.01358 |
| g__[Clostridium]_innocuum_group | 0.1542 | 0.09483 | 3.647 | 3.007 | 7.054 | 5.194 | 2.029 | 3.743 | 0.04545 | 0.1193 |
| g__Lachnospiraceae_NK4A136_group | 7.391 | 2.435 | 2.362 | 1.186 | 0.05452 | 0.1219 | 0.3899 | 0.8704 | 0.00129 | 0.01358 |
| g__norank_f__[Eubacterium]_coprostanoligenes_group | 5.892 | 1.508 | 0.528 | 0.7493 | 0.04413 | 0.07528 | 0.4242 | 0.6341 | 0.00072 | 0.01358 |
| g__Alistipes | 2.265 | 0.9206 | 2.755 | 1.026 | 0.7637 | 0.8082 | 0.7855 | 1.222 | 0.03013 | 0.0904 |
| g__Enterococcus | 0.00623 | 0.00873 | 0.7674 | 0.3499 | 1.287 | 0.6972 | 2.679 | 1.685 | 0.00247 | 0.01479 |
| g__norank_f__Lachnospiraceae | 2.576 | 1.283 | 0.3759 | 0.3699 | 0.1054 | 0.2357 | 0.08982 | 0.1895 | 0.01908 | 0.08001 |
| g__Lachnoclostridium | 1.398 | 0.8073 | 0.284 | 0.1864 | 0.1179 | 0.09392 | 0.4543 | 0.7124 | 0.04291 | 0.1193 |
| g__Adlercreutzia | 0.866 | 0.2162 | 0.1973 | 0.1986 | 0.08359 | 0.1096 | 0.9652 | 1.836 | 0.0011 | 0.01358 |
| g__Bifidobacterium | 0.09761 | 0.07221 | 0.7684 | 0.3389 | 0.2212 | 0.2612 | 0.7798 | 0.8586 | 0.02096 | 0.08001 |
| g__Marvinbryantia | 0.4237 | 0.1277 | 0.8723 | 0.509 | 0.03167 | 0.07082 | 0.4429 | 0.9845 | 0.00204 | 0.01479 |
| g__Acutalibacter | 0.5119 | 0.1693 | 0.2575 | 0.2691 | 0.03063 | 0.01625 | 0.2046 | 0.332 | 0.00385 | 0.02024 |
| g__norank_f__Eggerthellaceae | 0.05296 | 0.02986 | 0.1584 | 0.1017 | 0.00831 | 0.01438 | 0.03738 | 0.07498 | 0.02776 | 0.08968 |

**Note:** Data are expressed as Mean (%) and Standard Deviation (SD (%)). Statistical significance was assessed by one-way ANOVA based on raw relative abundance data. To control for the false discovery rate in multiple comparisons, *p*-values were adjusted using the Benjamini-Hochberg (BH) method (*p*_adjust).
